# Antifungal effects and active compounds of the leaf of *Allium mongolicum* Regel

**DOI:** 10.3389/fchem.2022.993893

**Published:** 2022-08-24

**Authors:** Huan Qu, Zhen Guo, Li Ma, Xiu Zhang, Haijun Ma, Yang Chen

**Affiliations:** ^1^ College of Biological Science and Engineering, North Minzu University, Yinchuan, Ningxia, China; ^2^ Ningxia Key Laboratory of Microbial Resources Development and Applications in Special Environment, Yinchuan, Ningxia, China; ^3^ Ningxia Grape and Wine Innovation Center, Yinchuan, Ningxia, China; ^4^ State Key Laboratory Breeding Base of Green Pesticide and Agricultural Bioengineering, Key Laboratory of Green Pesticide and Agricultural Bioengineering, Ministry of Education, Research and Development Center for Fine Chemicals, Guizhou University, Guiyang, China

**Keywords:** *Allium mongolicum*, antifungal activity, flavonoids, phenolic acids, tryptophan

## Abstract

Taking plant metabolites as material to develop new biological fungicides is still an important mission for pesticide development, and the preliminary study confirmed that *Allium mongolicum* showed a certain inhibitory effect on plant pathogens. In this study, the antifungal activity of extracts of *A. mongolicum* was studied and the compounds were isolated, purified, and identified by HPLC, NMR, and ESI-MS. The methanol extract of *A. mongolicum* exhibited certain inhibitory activity against almost all nine tested pathogens at concentration of 0.5 mg/ml. Sixteen compounds were isolated and purified from the extract, which were identified as nine flavonoids, six phenolic acids, and an amino acid. Among them, cinnamic acid derivatives **1**, **2**, and **3** and flavonoids **7**, **8**, **9**, and **13** were separated in *A. mongolicum* for the first time.

## 1 Introduction

Food and Agriculture Organization of the United Nations (FAO) estimates that about 25% of the world’s crops are affected by plant diseases every year (Xing, 2018), which lead to the yield losses as well as reduced quality of crop production (Gao et al., 2016; Liu and Wang, 2016) and secrete a variety of toxins and harmful metabolites (Guo et al., 2021). As is reported, 70%–80% of the plant diseases are caused by plant phytopathogenic fungi, such as *Magnaporthe oryzae*, *Fusarium graminearum*, *Valsa mali,* and *Botrytis cinerea*, which are hard to control (Qin et al., 2013). For a long time, chemical antimicrobial agents play an important role in inhibiting plant-pathogenic fungi and promoting agricultural production. However, the long-term use of chemical agents has gradually been limited by the problems of food safety, environmental pollution, and the tendency of pathogenic microorganisms to develop resistance to them (Kim and Hwang, 2007; Akhter et al., 2015; Zhang et al., 2018). Therefore, development of new compounds with effectively inhibiting activity is still an important mission (Kang, 2010). *Alliaceae* has become an important source for finding useful compounds due to its significant and extensive antifungal activity, some of which have been used for agricultural diseases (Ma et al., 2014). As representative plants of *Alliaceae*, *Allium sativum* and *Allium cepa* gained more attention and significantly inhibited many kinds of microorganisms (Nicastro et al., 2015; Martins et al., 2016; Chen et al., 2017). Especially, the fungicides with garlic oil and ethylicin as the main effective components were successfully used for control of agricultural fungal and bacterial diseases.

As a kind of characteristic xerophytic plant of *Allium*, *A. mongolicum* is widely distributed in the desert regions of northwest China and its leaves are used as a high-quality forage plant as well as a natural green uncontaminated food (Zhang et al., 2017). However, for a long time, studies of *A. mongolicum* have focused only on its morphology and geographic distribution, artificial cultivation and nutritional value, seed germination characteristics, and genetic diversity (Yang et al., 2008; Zhang, et al., 2014; Hu et al., 2017). Recent studies have found that *A. mongolicum* has broad-spectrum antimicrobial activity. The aqueous and ethanol extracts of *A. mongolicum* exhibited certain inhibitory effects on *Staphylococcus aureus* and *Escherichia coli* and showed greater tolerance to ultraviolet light and temperature. In the course of studying the effect of *A. mongolicum* on mutton quality, it found that volatile oil, polysaccharide, and flavonoids showed an inhibitory effect on animal-related foodborne pathogens (Sa et al., 2014; Muqier et al., 2017). As an important material for finding antimicrobial compounds, some monomeric compounds of flavonoid and ethyl cinnamate, dibutyl oxalate, and 2-hexenal from the essential oil of *A. mongolicum* were gained in recent years (Wang et al., 2012; Dong et al., 2015; Dong et al., 2020). However, the antimicrobial activity compounds of *A. mongolicum* have not been systematically explored at present. Therefore, comparatively systematic research about the inhibitory effect of extracts of *A. mongolicum* on common plant-pathogenic fungi was made based on the above in this article, and monomeric compound of extracts of *A. mongolicum* were isolated, purified, and identified, which would lay a foundation for the research and development of new botanical pesticides.

## 2 Materials and methods

### 2.1 Materials

*A. mongolicum* were collected from Luanjingtan in Alxa Zuoqi, Inner Mongolia in July (E 105°23′46.12″, N 37°51′36.42″, altitude 1,430 m). Healthy plants were selected, and aboveground parts were collected to be taken back to the laboratory. Tested plant pathogenic fungi: *Fusarium oxysporum*, *Exserohilum turcicum*, *Valsa mali*, *Fusarium graminearum*, *Botrytis cinerea*, *Alternaria solani,* and *Fusarium sulphureum* were provided by Northwest A&F University; *Fusarium solani* and *Corynespora cassiicola* were preserved and provided by Ningxia Key Laboratory of Microbial Resources Development and Applications in Special Environment.

### 2.2 Preparation of extracts of *A. mongolicum*

Plant extracts were prepared by solvent extraction with some modification (Prashith et al., 2015). The leaves of *A. mongolicum* were cut into small pieces and dried under shade for 7 days at 32–35°C, then powdered in a blender and stored in a sealed fresh-storage bottle away from light. Dry powder was separately extracted by soaking in 10times the volume of petroleum ether, dichloromethane, ethyl acetate, and methanol for 48 h. The extract was filtered out, and the same process is repeated three times. The filtrates were combined and concentrated to paste at 45–50°C by a rotary evaporator and stored in a refrigerator at 4°C for later used.

### 2.3 Antifungal activity assays of extracts of *A. mongolicum*

Antifungal activity of petroleum ether, dichloromethane, ethyl acetate, and methanol extracts was evaluated by the poisoned food technique (Liu et al., 2017). The extracts were prepared using acetone as the initial solvent carrier followed by dilution with PDA (at about 60°C) to produce the desired concentrations of 0.5 mg/ml, then poured into a sterile petri dish (9 cm in diameter) to make a medium-filled plate. After the medium was solidified, a mycelial disk (with a diameter of 4 mm) containing tested pathogenic fungi was placed in each medium plane. The PDA plates were incubated in the light-dark cycle at 28 ± 1°C for 5 days. PDA plates treated with an equal quantity of acetone were used as control. Each treatment was treated using three biological replicates. The mycelial growth inhibition was calculated by the formula.

Inhibition rate(%)=(C−T)/(C−4)×100

where C is the mycelial diameter (mm) of the control and T is the mycelial diameter (mm) under extract solution treatment.

### 2.4 Isolation, purification, and structure identification of compounds from *A. mongolicum*

*A. mongolicum* powder (6.4 kg) was extracted with methanol-water (4:1, v/v) for 1 h three times by using ultrasonic treatment. The extracts were combined and centrifuged, then discard precipitation; the filtrate was concentrated to paste at 45°C, dispersed in appropriate amount of water. The aqueous phase was extracted with petroleum ether and ethyl acetate in turn three times. The petroleum ether phase was discarded, and the ethyl acetate and water phase were retained.

The ethyl acetate phase was concentrated under reduced pressure at 45°C to obtain crude extract. The extract was isolated by normal-phase silica gel chromatography and then eluted with petroleum ether-ethyl acetate-methanol, ethyl acetate, and methanol-ethyl acetate fractions were collected. Ethyl acetate fraction was concentrated and dissolved in methanol, and separated based on high-performance liquid chromatography to gain the compounds of **1** (100 mg), **2** (90 mg), and **3** (125 mg). Methanol-ethyl acetate (5:1, v/v) fraction was concentrated and dissolved in methanol, separated using high-performance liquid chromatography (HPLC) to gain the compounds of **4** (240 mg) and **5** (40 mg). Methanol-ethyl acetate (1:1, v/v) fraction was concentrated and dissolved in methanol and separated using HPLC to gain the compounds of **6** (210 mg).

The aqueous phase was separated by AB-8 macroporous resin column chromatography with methanol-water as a mobile phase for gradient elution. Methanol-water (7:3, v/v) phase was collected, evaporated by rotary evaporator and dissolved in methanol for HPLC separation to gain 1, 2, 3, 4, and 5 fractions, fractions (1, 2, 3, 4, and 5) were further isolated through HPLC. Fraction 1 was eluted with methanol-water and acetonitrile-water to obtain compounds numbered **7** (60 mg), **8** (40 mg), **9** (30 mg), **10** (200 mg). Fraction 2 was eluted with methanol-water and acetonitrile-water to obtain compounds numbered **11** (10 mg), **12** (20 mg), **13** (40 mg). Fraction 3 was concentrated to remove some solvents and the compound **14** (1,500 mg) separated out with standing for 24 h. Fraction 5 was eluted with methanol-water and acetonitrile-water to obtain compounds numbered **15** (60 mg), **6** (905 mg), and **16**(70 mg).

### 2.5 Compound structure identification

The mass spectrum, ^1^H-NMR (400 MHz), and ^13^C-NMR (100 MHz) spectra of the compounds were determined. The chemical structure of the compounds was identified according to spectroscopic data.

## 3 Results and discussion

### 3.1 Results

#### 3.1.1 Toxicity determination of extracts of *A. mongolicum*

The extract of *A. mongolicum* showed different degrees of inhibition on the mycelial growth of nine different plant-pathogenic fungi (Table 1). Among them, the inhibitory effects of all tested extracts on *Fusarium solani* and *Fusarium graminearum* were better, and the inhibition rate was about over 30%. Generally, the inhibitory effects of petroleum ether extract and methanol extract were higher than that of dichloromethane extract and ethyl acetate extract, and petroleum ether extract and methanol extract almost showed inhibitory activity on all tested pathogens. Based on the former results, the methanol extract with the yield of 23.31% was much higher than that of petroleum ether extract (4.27%), as well as, petroleum ether extract contained more pigment. Considering the polarity of the solvent, the methanol extract is richer in types of compounds compared with petroleum ether extract. Therefore, methanol was selected as the solvent in the subsequent extract experiment.

**TABLE 1:** Toxicity test results of extracts of *A. mongolicum* against nine plant-pathogenic fungi at a concentration of 0.5 mg/ml (96 h of incubation).

Plant-pathogenic fungi	Inhibition rate (%; mean ± SD; *N* = 3)^a^			
	Petroleum ether extract	Dichloromethane extract	Ethyl acetate extract	Methanol extract
*Fusarium solani*	36.61 ± 0.23	36.96 ± 0.11	34.46 ± 0.04	30.36 ± 0.20
*Valsa mali*	42.63 ± 0.20	20.59 ± 0.12	30.88 ± 0.13	32.35 ± 0.18
*Fusarium oxysporum*	44.77 ± 0.18	15.95 ± 0.12	12.95 ± 0.13	43.55 ± 0.25
*Fusarium sulphureum*	47.94 ± 0.19	5.39 ± 0.12	17.41 ± 0.12	19.21 ± 0.21
*Botrytis cinerea*	47.33 ± 0.23	22.81 ± 0.25	11.25 ± 0.15	38.73 ± 0.35
*Alternaria solani*	26.67 ± 0.13	10.00 ± 0.42	6.67 ± 0.12	33.33 ± 0.20
*Fusarium graminearum*	27.16 ± 0.16	45.31 ± 0.16	37.04 ± 0.12	33.70 ± 0.18
*Exserohilum turcicum*	3.45 ± 0.43	13.79 ± 0.24	25.29 ± 0.08	11.49 ± 0.16
*Corynespora cassiicola*	48.70 ± 0.17	5.44 ± 0.20	42.55 ± 0.15	13.24 ± 0.09
CK	0.00 ± 0.00	0.00 ± 0.00	0.00 ± 0.00	0.00 ± 0.00

a Values are the mean ± SE of three replicates.

#### 3.1.2 Chemical structure identification of compounds from *A. mongolicum*

**FIGURE 1:**
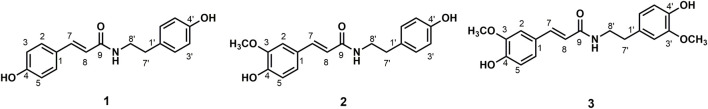
Chemical structure of compounds **1**, **2**, and **3**

Data for compound **1**: gray powder, m. p. 212–213°C; ESI-MS: Calcd for C_17_H_17_NO_3_ ([M + H]^+^), 284.16; ^1^H NMR (400 MHz, Methanol-*d*_4_) *δ*: 7.44 (d, *J* = 15.7 Hz, 1H, H-7), 7.39 (d, *J* = 8.6 Hz, 2H, H-2, 6), 7.09–7.02 (m, 2H, H-2′, 6′), 6.82–6.76 (m, 2H, H-3, 5), 6.75–6.66 (m, 2H, H-3′, 5′), 6.38 (d, *J* = 15.7 Hz, 1H,H-8), 3.46 (t, *J* = 7.4 Hz, 2H, H-8′), 2.75 (t, *J* = 7.4 Hz, 2H, H-7′); ^13^C NMR (100 MHz, Methanol-*d*_4_) *δ*: 169.25 (C-9), 160.49 (C-4), 156.91 (C-4′), 141.77 (C-7), 131.33 (C-1′), 130.72 (C-2′, 6′), 130.54 (C-2, 6), 127.75 (C-1), 118.47 (C-8), 116.72 (C-3′, 5′), 116.27 (C-3, 5), 42.53 (C-8′), and 35.80 (C-7′). According to the abovementioned data and literature (Kim and Lee, 2003), compound **1** was identified as *trans*-*N*-*p*-coumaroyl tyramine and its molecular formula is shown in Figure 1. MS diagrams and ^1^H-NMR and ^13^C-NMR spectra were shown in Supplementary Figures S1–S3, respectively.

Data for compound **2**: white powder, m. p. 123–125°C; ESI-MS: Calcd for C_18_H_19_NO_4_([M + H]^+^), 314.16; ^1^H NMR (400 MHz, Methanol-*d*_4_) *δ*: 7.47–7.40 (m, 1H, H-7), 7.11 (d, *J* = 1.9 Hz, 1H, H-2), 7.07–7.04 (m, 2H, H-2′, 6′), 7.02 (dd, *J* = 8.2, 2.0 Hz, 1H, H-6), 6.81 (s, 1H, H-5), 6.74–6.70 (m, 2H, H-3′, 5′), 6.40 (d, *J* = 15.7 Hz, 1H, H-8), 3.88 (s, 3H, H-3), 3.47 (dd, *J* = 8.0, 6.7 Hz, 2H, H-8′), 2.75 (t, *J* = 7.3 Hz, 2H, H-7′); ^13^C NMR (100 MHz, Methanol-*d*_4_) *δ*: 169.18 (C-9), 156.92 (C-4′), 149.82 (C-3), 149.29 (C-4), 142.01 (C-7), 131.32 (C-1′), 130.72 (C-2′, 6′), 128.31 (C-1), 123.22 (C-6), 118.80 (C-8), 116.47 (C-3′, 5′), 116.28 (C-5), 111.59 (C-2), 56.41 (OCH_3_-3), 42.52 (C-8′), and 35.79 (C-7′). According to the abovementioned data and literature (Jiang and Ying, 2017), compound **2** was identified as *N*-*trans*-feruloyl tyramine and its molecular formula is shown in Figure 1. MS diagrams and ^1^H-NMR and ^13^C-NMR spectra were shown in Supplementary Figures S4–S6, respectively.

Data for compound **3**: steel gray solid, m. p. 132–134°C; ESI-MS: Calcd for C_19_H_21_NO_5_([M + H]^+^), 344.19; ^1^H NMR (400 MHz, Methanol-*d*_4_) *δ*: 7.44 (d, *J* = 15.7 Hz, 1H, H-7), 7.11 (d, *J* = 1.9 Hz, 1H, H-6), 7.02 (dd, *J* = 8.2, 2.0 Hz, 1H, H-2), 6.84–6.74 (m, 2H,H-2′, 6′), 6.72 (d, *J* = 8.0 Hz, 1H, H-3), 6.66 (dd, *J* = 8.0, 1.9 Hz, 1H), 6.41 (d, *J* = 15.7 Hz, 1H, H-8), 3.87 (s, 3H, H-5), 3.82 (s, 3H, H-3′), 3.49 (dd, *J* = 8.0, 6.7 Hz, 2H, H-8′), 2.77 (t, *J* = 7.3 Hz, 2H, H-7′); ^13^C NMR (100 MHz, Methanol-*d*_4_) *δ*: 169.19 (C-9), 149.84 (C-4), 149.29 (C-3), 148.95 (C-3′), 146.05 (C-4′), 142.02 (C-7), 132.05 (C-1′), 128.29 (C-1), 123.19 (C-6), 122.26 (C-6′), 118.80 (C-8), 116.48 (C-5), 116.21 (C-5′), 113.49 (C-2′), 111.59 (C-2), 56.40 (OCH_3_-3), 56.37 (OCH_3_-3′), 42.46 (C-8′), and 36.20 (C-7′). According to the abovementioned data and literature (Jiang and Ying, 2017), compound **3** was identified as *N*-*trans*-feruloyl-3-methoxy tyramine and its molecular formula is shown in Figure 1. MS diagrams and ^1^H-NMR and ^13^C-NMR spectra were shown in Supplementary Figures S7–S9, respectively.

**FIGURE 2:**
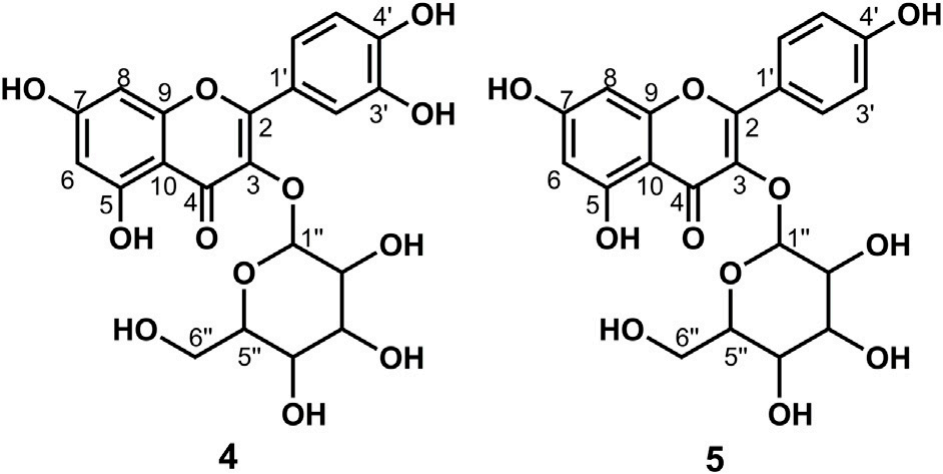
Chemical structure of compounds **4** and **5**

Data for compound **4**: pale yellow powder, m. p. 179–182°C; ESI-MS: Calcd for C_21_H_20_O_12_ ([M + H]^+^), 465.12; ^1^H NMR (400 MHz, DMSO-*d*_6_) *δ*: 12.63 (s, 1H, H-5), 10.91 (s, 1H,H-4′), 9.72 (s, 1H, H-7), 9.21 (s, 1H, H-3′), 7.62–7.52 (m, 2H, H-2′, 6′), 6.89–6.76 (m, 1H, H-5′), 6.42 (d, *J* = 2.1 Hz, 1H, H-8), 6.21 (d, *J* = 2.0 Hz, 1H, H-6), 5.46 (d, *J* = 7.4 Hz, 1H, H-1″), 5.27 (d, *J* = 3.8 Hz, 1H), 5.07–5.02 (m, 1H), 4.94 (d, *J* = 3.6 Hz, 1H), 4.24 (t, *J* = 5.7 Hz, 1H), 3.59 (dd, *J* = 11.5, 4.4 Hz, 1H), 3.30–3.20 (m, 3H), 3.20–3.05 (m, 2H, H-6″); ^13^C NMR (100 MHz, DMSO-*d*_6_) *δ*: 177.45 (C-4), 164.15 (C-7), 161.24 (C-5), 156.32 (C-9), 156.20 (C-2), 148.47 (C-4′), 144.82 (C-3′), 133.35 (C-3), 121.60 (C-6′), 121.16 (C-1′), 116.21 (C-2′), 115.22 (C-5′), 103.98 (C-10), 100.91 (C-1″), 98.67 (C-6), 93.51 (C-8), 77.55 (C-3″), 76.52 (C-5″), 74.10 (C-2″), 69.95 (C-4″), and 60.98 (C-6″). According to the abovementioned data and literature (Dong et al., 2016), compound **4** was identified as Isoquercitrin and its molecular formula is shown in Figure 2. MS diagrams and ^1^H-NMR and ^13^C-NMR spectra were shown in Supplementary Figures S10–S12, respectively.

Data for compound **5**: pale yellow powder, m. p. 196–198°C; ESI-MS: Calcd for C_21_H_20_O_11_([M + H]^+^), 449.14; ^1^H NMR (400 MHz, DMSO-*d*_6_) *δ*: 12.61 (s, 1H, H-5), 10.89 (s, 1H, H-4′), 10.18 (s, 1H, H-7), 8.08–8.00 (m, 2H, H-2′, 6′), 6.97–6.85 (m, 2H, H-3′, 5′), 6.44 (d, *J* = 2.1 Hz, 1H, H-8), 6.22 (d, *J* = 2.1 Hz, 1H, H-6), 5.45 (d, *J* = 7.3 Hz, 1H, H-1″), 5.31 (d, *J* = 4.3 Hz, 1H), 5.02 (d, *J* = 4.4 Hz, 1H), 4.92 (d, *J* = 3.7 Hz, 1H), 4.23 (t, *J* = 5.6 Hz, 1H), 3.57 (dd, *J* = 11.6, 5.0 Hz, 1H), 3.28–3.15 (m, 3H), 3.14–3.04 (m, 2H, H-6″); ^13^C NMR (100 MHz, DMSO-*d*_6_) *δ*: 177.47 (C-4), 164.15 (C-7), 161.21 (C-5), 159.96 (C-4′), 156.38 (C-2), 156.26 (C-9), 133.21 (C-3), 130.86 (C-2′, C-6′), 120.88 (C-1′), 115.10 (C-3′, 5′), 104.00 (C-10), 100.91 (C-1″), 98.69 (C-6), 93.64 (C-8), 77.47 (C-3″), 76.44 (C-5″), 74.21 (C-2″), 69.91 (C-4″), and 60.85 (C-6″). According to the abovementioned data and combined with the literature (Dong et al., 2015), compound **5** was identified as Kaempferol-3-*O*-glucoside and its molecular formula is shown in Figure 2. MS diagrams and ^1^H-NMR and ^13^C-NMR spectra were shown in Supplementary Figures S13–S15, respectively.

**FIGURE 3:**
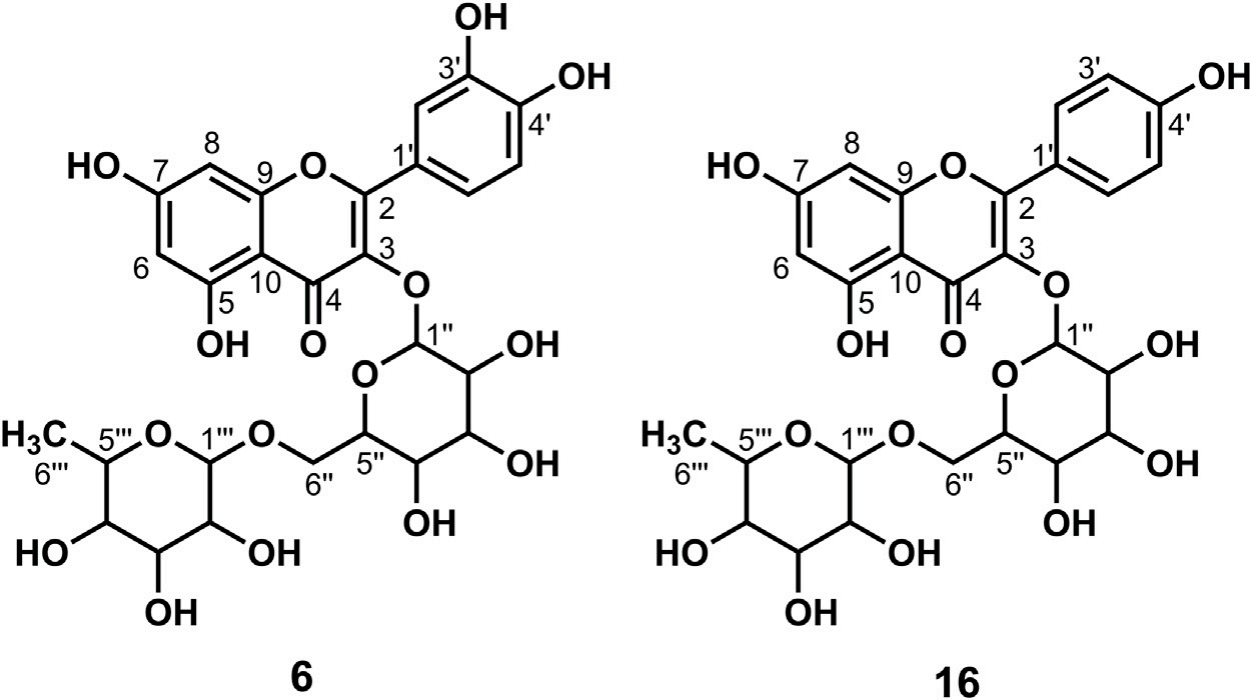
Chemical structure of compounds **6** and **16**.

Data for compound **6**: pale yellow powder, m. p. 175–177°C; ESI-MS: Calcd for C_27_H_30_O_16_ ([M + H]^+^), 611.19; ^1^H NMR (400 MHz, DMSO-*d*_6_) *δ*: 12.59 (s, 1H, H-5), 10.81 (s, 1H,H-4′), 9.61 (s, 1H, H-7), 9.16 (s, 1H, H-3′), 7.54 (d, *J* = 7.5 Hz, 2H, H-2′, 6′), 6.85–6.84 (m, 1H, H-5′), 6.39 (d, *J* = 2.1 Hz, 1H, H-8), 6.20 (d, *J* = 2.1 Hz, 1H,H-6), 5.39–5.30 (m, 1H, 1″), 5.2 (s, 1H), 5.05 (s, 1H), 4.39 (d, *J* = 1.6 Hz, 2H), 4.34 (s, 1H), 3.71 (d, *J* = 10.6 Hz, 1H), 3.40 (dd, *J* = 3.5, 1.6 Hz, 1H), 3.31–3.20 (m, 7H), 3.08 (t, *J* = 9.2 Hz, 3H), 1.00 (d, *J* = 6.2 Hz, 3H, H-6‴); ^13^C NMR (100 MHz, DMSO-*d*_6_) *δ*: 177.35 (C-4), 164.07 (C-7), 161.20 (C-5), 156.57 (C-9), 156.40 (C-2), 148.39 (C-4′), 144.73 (C-3′), 133.30 (C-3), 121.56 (C-6′), 121.16 (C-1′), 116.25 (C-2′), 115.21 (C-5′), 103.94 (C-10), 101.18 (C-1″), 100.72 (C-1‴), 98.65 (C-6), 93.55 (C-8), 76.45 (C-3″), 75.90 (C-5″), 74.06 (C-2″), 71.84 (C-4‴), 70.55 (C-3‴), 70.35 (C-2‴), 69.99 (C-4″), 68.21 (C-5‴), 66.97 (C-6″), and 17.70 (C-6‴). According to the abovementioned data and literature (Dong et al., 2016), compound **6** was identified as Rutin and its molecular formula is shown in Figure 3. MS diagrams and ^1^H-NMR and ^13^C-NMR spectra were shown in Supplementary Figures S16–S18, respectively.

**FIGURE 4:**
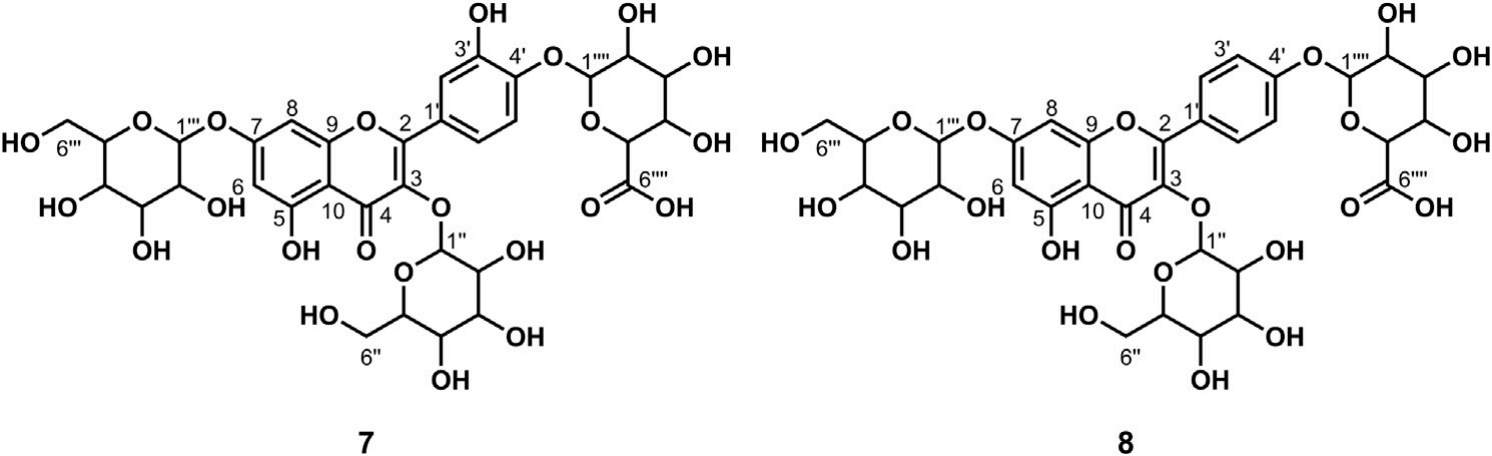
Chemical structure of compounds **7** and **8**

Data for compound **7**: pale yellow powder, m. p. 187–189°C; ESI-MS: Calcd for C_33_H_38_O_23_([M + H]^+^), 803.05; ^1^H NMR (400 MHz, DMSO-*d*_6_) *δ*: 12.56 (s, 1H, H-5), 8.93 (s, 1H, H-3′), 7.68 (d, *J* = 2.2 Hz, 1H, H-2′), 7.64 (dd, *J* = 8.6, 2.3 Hz, 1H, H-6′), 7.23 (d, *J* = 8.7 Hz, 1H, H-5′), 6.85 (d, *J* = 2.1 Hz, 1H, H-8), 6.46 (d, *J* = 2.1 Hz, 1H, H-6), 5.56–5.44 (m, 4H), 5.30 (t, *J* = 2.4 Hz, 1H), 5.23 (d, *J* = 7.3 Hz, 2H), 5.09 (s, 3H), 4.97 (s, 1H), 4.87 (d, *J* = 7.1 Hz, 1H, H-1‴), 4.63 (t, *J* = 5.7 Hz, 1H), 4.31 (t, *J* = 5.8 Hz, 1H), 3.92 (d, *J* = 7.9 Hz, 1H), 3.77–3.70 (m, 2H), 3.59 (dd, *J* = 11.3, 5.0 Hz, 2H), 3.49 (d, *J* = 6.1 Hz, 2H), 3.26–3.15 (m, 7H), 3.10 (d, *J* = 4.1 Hz, 3H); ^13^C NMR (100 MHz, DMSO-*d*_6_) *δ*: 177.75 (C-4), 172.00 (C-6‴'), 162.68 (C-7), 160.85 (C-5), 156.07 (C-2), 156.07 (C-9), 147.67 (C-4′), 146.19 (C-3′), 134.16 (C-3), 124.33 (C-1′), 121.06 (C-6′), 116.64 (C-2′), 115.45 (C-5′), 105.81 (C-10), 101.48 (C-1‴), 100.65 (C-1″), 99.35 (C-1‴'), 99.20 (C-6), 94.33 (C-8),77.69 (C-5″), 77.26 (C-5‴), 76.50 (C-3″), 75.94 (C-3‴'), 75.83 (C-3‴), 74.85 (C-5‴'), 74.12 (C-2″), 73.26 (C-2‴), 72.80 (C-2‴'), 71.49 (C-4‴'), 70.00 (C-4″), 69.78 (C-4‴), 60.97 (C-6″), and 60.72 (C-6‴). According to the abovementioned data and literature (Fossen et al., 2006; Dong et al., 2020), compound **7** was identified as Quercetin-3,7-*O*-diglucoside-4′-*O*-glucuronide and its molecular formula is shown in Figure 4. MS diagrams and ^1^H-NMR and ^13^C-NMR spectra were shown in Supplementary Figures S19–S21, respectively.

Data for compound **8**: pale yellow powder, m. p. 185–187°C; ESI-MS: Calcd for C_33_H_38_O_22_ ([M + H]^+^), 787.29; ^1^H NMR (400 MHz, DMSO-*d*_6_) *δ:*12.56 (s, 1H, H-5), 8.15 (d, *J* = 8.9 Hz, 2H, H-2′, 6′), 7.18 (d, *J* = 8.9 Hz, 2H, H-3′, 5′), 6.87 (d, *J* = 2.2 Hz, 1H, H-8), 6.47 (d, *J* = 2.2 Hz, 1H, H-6), 5.52 (d, *J* = 4.7 Hz, 1H), 5.48 (d, *J* = 7.3 Hz, 1H, H-1″), 5.35 (dd, *J* = 11.6, 4.5 Hz, 2H), 5.28 (d, *J* = 6.7 Hz, 2H), 5.12 (s, 1H), 5.09–5.01 (m, 3H), 4.97 (d, *J* = 4.2 Hz, 1H), 4.65–4.52 (m, 1H), 4.31 (t, *J* = 5.8 Hz, 1H), 4.02 (d, *J* = 9.3 Hz, 1H), 3.71 (dd, *J* = 11.5, 4.7 Hz, 1H), 3.57 (dd, *J* = 11.7, 5.7 Hz, 2H), 3.52–3.47 (m, 3H), 3.30–3.05 (m, 11H);^13^C NMR (100 MHz, DMSO-*d*_6_) *δ*: 177.77 (C-4), 170.23 (C-6‴'), 162.54 (C-7), 160.91 (C-5), 159.38 (C-4′), 156.24 (C-9), 156.13 (C-2), 134.07 (C-3), 130.66 (C-2′, 6′), 123.58 (C-1′), 115.85 (C-3′, 5′), 105.91 (C-10), 100.76 (C-1″), 99.97 (C-1‴), 99.33 (C-6), 99.11 (C-1‴'), 94.39 (C-8), 77.62 (C-5″), 77.11 (C-5‴), 76.55 (C-3‴), 76.45 (C-3″), 75.73 (C-3‴'), 75.27 (C-5‴'), 74.20 (C-2″), 73.23 (C-2‴), 72.77 (C-2‴'), 71.28 (C-4‴'), 69.94 (C-4″), 69.66 (C-4‴), 60.88 (C-6″), and 60.66 (C-6‴). According to the abovementioned data and literature (Dong et al., 2020), compound **8** was identified as Kaempferol-3,7-*O*-diglucoside-4′-*O*-glucuronide and its molecular formula is shown in Figure 4. MS diagrams and ^1^H-NMR and ^13^C-NMR spectra were shown in Supplementary Figures S22–S24, respectively.

**FIGURE 5:**
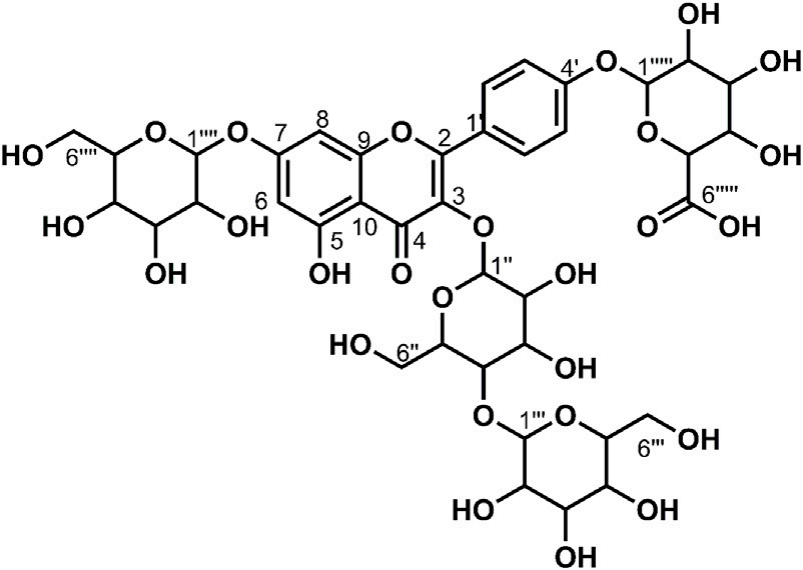
Chemical structure of compound **9**.

Data for compound **9**: pale yellow powder, m. p. 181–182°C; ESI-MS: Calcd for C_39_H_48_O_27_([M + H]^+^), 949.52; ^1^H NMR (400 MHz, DMSO-*d*_6_) *δ*: 12.54 (s, 1H, H-5), 8.18–8.06 (m, 2H, H-2′, 6′), 7.22–7.12 (m, 2H, H-3′, 5′), 6.86 (d, *J* = 2.1 Hz, 1H, H-8), 6.47 (d, *J* = 2.1 Hz, 1H, H-6), 5.57–5.42 (m, 4H), 5.36 (d, *J* = 4.6 Hz, 1H), 5.22 (dd, *J* = 12.1, 6.0 Hz, 4H), 5.12 (s, 1H, H-1‴''), 5.08–4.96 (m, 5H), 4.76 (d, *J* = 1.9 Hz, 1H), 4.64–4.55 (m, 2H), 4.40 (t, *J* = 5.8 Hz, 1H), 4.27 (d, *J* = 7.8 Hz, 1H, H-1‴), 3.93 (d, *J* = 8.6 Hz, 1H), 3.76–3.68 (m, 3H), 3.67–3.59 (m, 2H), 3.58–3.43 (m, 5H), 3.26–3.11 (m, 7H), 3.07 (d, *J* = 9.5 Hz, 1H), 3.06–2.99 (m, 2H); ^13^C NMR (100 MHz, DMSO-*d*_6_) *δ*: 177.67 (C-4), 170.87 (C-6‴''), 162.69 (C-7), 160.86 (C-5), 159.39 (C-4′), 156.29 (C-2), 156.11 (C-9), 134.00 (C-3), 130.64 (C-2′, 6′), 123.50 (C-1′), 115.87 (C-3′, 5′), 105.84 (C-10), 103.09 (C-1‴), 100.51 (C-1″), 99.95 (C-1‴''), 99.40 (6), 99.21 (C-1‴'), 94.45 (C-8), 80.05 (C-4″), 77.09 (C-5‴'), 76.78 (C-5‴), 76.53 (C-3‴'), 76.44 (C-3‴),75.89 (C-3‴''), 75.50 (C-5″),74.74 (C-3″), 73.93 (C-5‴''), 73.26 (C-2‴, C-2‴'), 73.22 (C-2″), 72.80 (C-2‴''), 71.43 (C-4‴''), 70.03 (C-4‴), 69.64 (C-4‴'), 61.01 (C-6‴), 60.66 (C-6‴'), and 60.16 (C-6″). According to the abovementioned data and literature (Dong et al., 2020), compound **9** was identified as Kaempferol-3-*O*-gentiobiose-7-*O*-glucose-4′-*O*-glucuronide and its molecular formula is shown in Figure 5. MS diagrams and ^1^H-NMR and ^13^C-NMR spectra were shown in Supplementary Figures S25–S27, respectively.

**FIGURE 6:**
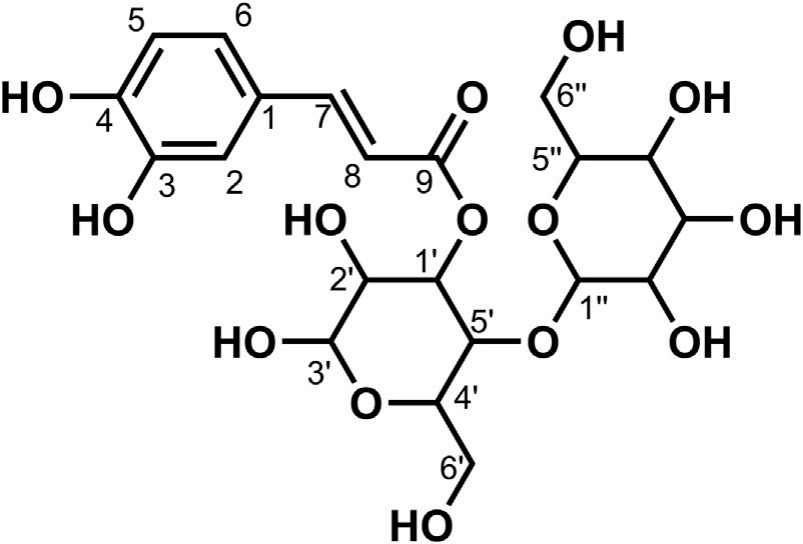
Chemical structure of compound **10**.

Data for compound **10**: pale yellow crystal solid, m. p. 140–145°C; ESI-MS: Calcd forC_21_H_28_O_14_ ([M-H]^-^), 503.20; ^1^H NMR (400 MHz, DMSO-*d*_6_) *δ*: 7.55 (d, *J* = 15.9 Hz, 1H, H-7), 7.40 (d, *J* = 15.9 Hz, 1H, H-7), 7.06 (d, *J* = 2.1 Hz, 1H, H-2), 7.07–7.00 (m, 1H, H-2), 7.04–6.98 (m, 1H, H-6), 6.95 (dd, *J* = 8.2, 2.1 Hz, 1H, H-6), 6.76 (dd, *J* = 8.2, 6.0 Hz, 2H, H-5), 6.27 (d, *J* = 15.9 Hz, 1H, H-8), 6.16 (d, *J* = 15.9 Hz, 1H, H-8), 5.60–5.52 (m, 1H, H-3′), 5.15 (d, *J* = 3.4 Hz, 1H), 5.16–4.35 (m, 16H), 3.70–3.32 (m, 15H), 3.25–2.85 (m, 13H), 2.06 (s, 1H); ^13^C NMR (100 MHz, DMSO-*d*_6_) *δ*: 167.83 (C-9), 164.95 (C-9), 148.58 (C-4), 148.11 (C-4), 146.17 (C-7), 145.54 (C-3), 144.54 (C-3), 125.68 (C-1), 125.56 (C-1), 121.58 (C-6), 121.09 (C-6), 115.73 (C-5), 115.09 (C-5), 114.88 (C-2), 114.61 (C-2), 113.53 (C-8), 105.09 (C-1″), 104.56 (C-1″), 92.46 (C-1′), 91.44 (C-1′), 82.22 (C-2′), 81.66 (C-2′), 77.63 (C-5″), 76.83 (C-3″), 76.72 (C-3″), 76.38 (C-5′), 76.18 (C-5′), 75.75 (C-4′), 74.56 (C-3′), 73.87 (C-3′), 71.83 (C-2″), 71.68 (C-2″), 70.18 (C-2′), 70.08 (C-2′), 69.46 (C-4″), 69.17 (C-4″), 61.20 (C-6′), 61.11 (C-6″), 60.46 (C-6′), and 60.41 (C-6″). According to the abovementioned data and literature (Duan, et al., 2012; Dong et al., 2020), compound **10** was identified as 1-Caffeoyl gentiobioside (two kinds of sugar configurations: *α* and *β*) and its molecular formula is shown in Figure 6. MS diagrams and ^1^H-NMR and ^13^C-NMR spectra were shown in Supplementary Figures S27–S30, respectively.

**FIGURE 7:**
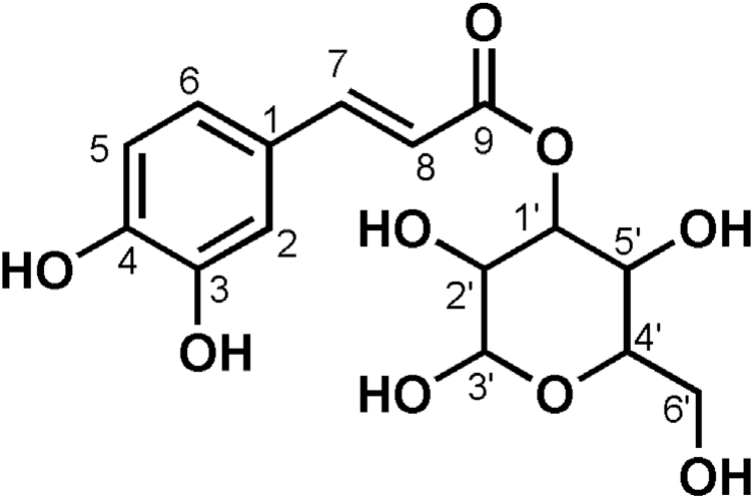
Chemical structure of compound **11**.

Data for compound **11**: white powder, m. p. 142–144°C; ESI-MS: Calcd for C_15_H_18_O_9_([M-H]^-^), 341.17; ^1^H NMR (400 MHz, DMSO-*d*_6_) *δ*: 7.55 (d, *J* = 15.8 Hz, 1H, H-7), 7.08 (d, *J* = 2.1 Hz, 1H, H-2), 7.01 (dd, *J* = 8.2, 2.0 Hz, 1H, H-6), 6.78 (d, *J* = 8.2 Hz, 1H, H-5), 6.26 (d, *J* = 15.9 Hz, 1H,H-8), 5.45 (d, *J* = 7.9 Hz, 1H), 5.28 (s, 1H), 5.06 (s, 2H,H-6′), 4.58 (s, 1H), 3.69–3.62 (m, 2H), 3.31–3.10 (m, 6H); ^13^C NMR (100 MHz, DMSO-*d*_6_) *δ*: 165.39 (C-9), 148.81 (C-4), 146.47 (C-7), 145.70 (C-3), 125.42 (C-1), 121.74 (C-6), 115.86 (C-5), 114.91 (C-8), 113.41 (C-2), 94.31 (C-1′), 77.86 (C-5′), 76.52 (C-3′), 72.56 (C-2′), 69.60 (C-4′), and 60.67 (C-6′). According to the abovementioned data and literature (Dong et al., 2020), compound **11** was identified as 1-Caffeoyl glucoside and its molecular formula is shown in Figure 7. MS diagrams and ^1^H-NMR and ^13^C-NMR spectra were shown in Supplementary Figures S31–S33, respectively.

**FIGURE 8:**
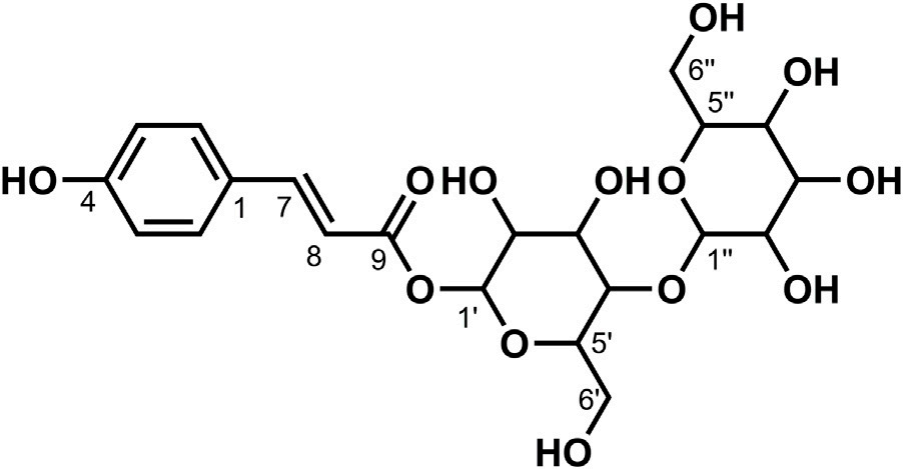
Chemical structure of compound **12**.

Data for compound **12**: pale yellow crystal solid, m. p. 118–119°C; ESI-MS: Calcd for C_21_H_28_O_13_ ([M-H]^-^), 487.21; ^1^H NMR (400 MHz, DMSO-*d*_6_) *δ*: 10.06 (s, 1H, H-4), 7.63 (d, *J* = 15.9 Hz, 1H, H-7), 7.59–7.54 (m, 2H,H-2, 6), 6.84–6.74 (m, 2H, H-3, 5), 6.40 (d, *J* = 16.0 Hz, 1H, H-8), 5.61–5.51 (m, 1H), 5.42 (s, 1H), 5.14 (s, 2H), 4.84 (s, 2H), 4.59 (s, 1H), 4.43 (d, *J* = 7.8 Hz, 1H), 3.67 (dd, *J* = 12.1, 2.0 Hz, 1H), 3.59–3.43 (m, 5H), 3.25–3.13 (m, 3H), 3.13–3.06 (m, 3H), 2.95 (dd, *J* = 8.9, 7.8 Hz, 1H); ^13^C NMR (100 MHz, DMSO-*d*_6_) *δ*: 165.01 (C-9), 160.00 (C-4), 145.74 (C-7), 130.40 (C-2), 130.04 (C-6), 125.13 (C-1), 115.78 (C-3), 11 5.31 (C-5), 113.76 (C-8), 104.57 (C-1″), 92.46 (C-1′), 81.67 (C-2′), 77.65 (C-5′), 76.76 (C-5″), 76.19 (C-3″), 75.77 (C-3′), 74.55 (C-2″), 69.52 (C-4″), 69.18 (C-4′), 60.50 (C-6″), and 60.41 (C-6′). According to the abovementioned data and literature (Dong et al., 2020), compound **12** was identified as 1-*p*-Coumaroyl gentiobioside and its molecular formula is shown in Figure 8. MS diagrams and ^1^H-NMR and ^13^C-NMR spectra were shown in Supplementary Figures S34–S36, respectively.

**FIGURE 9:**
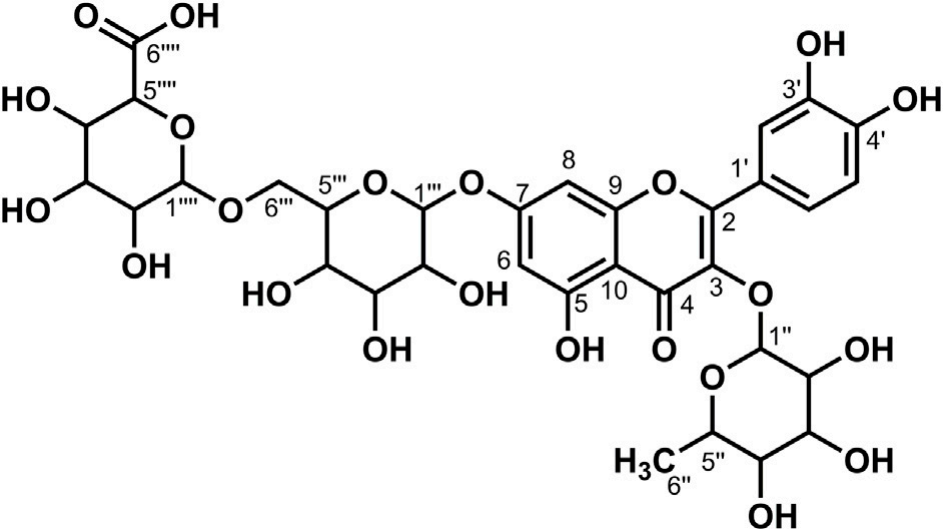
Chemical structure of compound **13**.

Data for compound **13**: pale yellow powder, m. p. 207–210°C; ESI-MS: Calcd for C_33_H_38_O_22_ ([M + H]^+^), 787.26; ^1^H NMR (400 MHz, DMSO-*d*_6_) *δ*: 12.62 (s, 1H, H-5), 9.74 (s, 1H, H-4′), 9.19 (s, 1H, H-3′),7.60–7.52 (m, 2H, H-2′, 6′), 6.86 (d, *J* = 8.2 Hz, 1H, H-5′), 6.74 (d, *J* = 2.1 Hz, 1H, H-8), 6.45 (d, *J* = 2.1 Hz, 1H, H-6), 5.48 (s, 1H, H-1′), 5.35 (d, *J* = 7.1 Hz, 1H), 5.25 (dd, *J* = 10.5, 5.6 Hz, 2H), 5.12–5.03 (m, 2H), 4.39 (d, *J* = 1.6 Hz, 1H), 3.94 (d, *J* = 8.8 Hz, 1H), 3.71 (d, *J* = 10.1 Hz, 1H), 3.36–3.21 (m, 15H), 3.17 (s, 1H), 3.12–3.03 (m, 2H, H-6‴), 0.98 (d, *J* = 6.2 Hz, 3H, H-6″); ^13^C NMR (100 MHz, DMSO-*d*_6_) *δ*: 177.51 (C-4), 170.68 (C-6‴'), 162.45 (C-7), 160.84 (C-5), 157.31 (C-2), 156.06 (C-9), 148.61 (C-4′), 144.77 (C-3′), 133.55 (C-3), 121.65 (C-1′), 121.03 (C-6′), 116.47 (C-2′), 115.21 (C-5′), 105.71 (C-10), 101.05 (C-1‴'), 100.83 (C-1″), 99.23 (C-6, 1‴), 94.45 (C-8), 76.44 (C-3‴), 75.94 (C-5‴), 75.81 (C-5‴'), 74.93 (C-3‴'), 74.02 (C-2‴), 72.85 (C-2‴'), 71.82 (C-4‴'), 71.41 (C-4″), 70.66 (C-2″), 70.35 (C-3″), 70.14 (C-4‴), 68.19 (C-5″), 67.23 (C-6‴), and 17.70 (C-6″). According to the abovementioned data and literature (Dong et al., 2020), compound **13** was identified as Quercetin-3-*O*-glucose-7-*O*-glucose (1→6)-*O*-glucuronide and its molecular formula is shown in Figure 9. MS diagrams and ^1^H-NMR and ^13^C-NMR spectra were shown in Supplementary Figures S37–S39, respectively.

**FIGURE 10:**
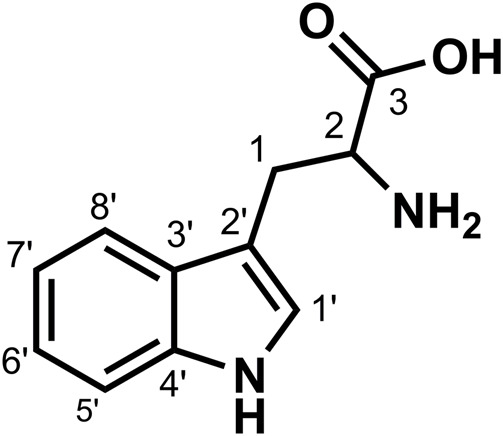
Chemical structure of compound **14**.

Data for compound **14**: white powder, m. p. 211–213°C; ESI-MS: Calcd for C_11_H_12_N_2_O_2_ ([M + H]^+^), 205.15; ^1^H NMR (400 MHz, DMSO-*d*_6_) *δ*: 11.04 (d, *J* = 2.5 Hz, 1H, H-3), 7.57 (d, *J* = 7.9 Hz, 1H, H-8′), 7.35 (d, *J* = 8.1 Hz, 1H, H-5′), 7.25 (d, *J* = 2.5 Hz, 1H, H-6′), 7.10–7.01 (m, 1H, H-1′), 7.01–6.92 (m, 1H, H-7′), 3.51–3.47 (m, 1H), 3.32 (dd, *J* = 15.1, 4.2 Hz, 1H), 3.02 (dd, *J* = 15.1, 8.6 Hz, 1H); ^13^C NMR (100 MHz, DMSO-*d*_6_) *δ*: 170.48 (C-3), 136.34 (C-4′), 127.30 (C-3′), 124.12 (C-1′), 120.79 (C-7′), 118.34 (C-6′), 118.20 (C-8′), 111.32 (C-5′), 109.56 (C-2′), 54.74 (C-2), and 27.11 (C-1). According to the abovementioned data, compound **14** was identified as tryptophan and its molecular formula is shown in Figure 10. MS diagrams and ^1^H-NMR and ^13^C-NMR spectra were shown in Supplementary Figures S40–S42, respectively.

**FIGURE 11:**
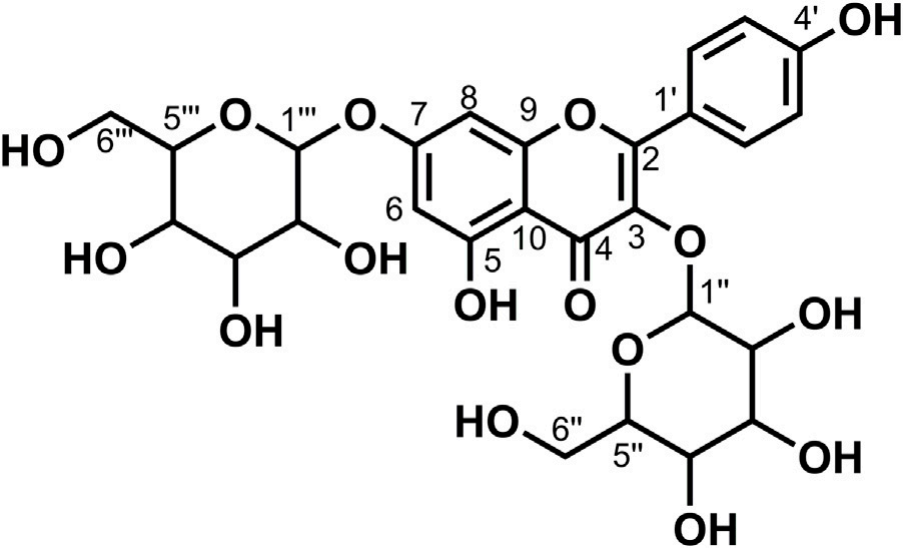
Chemical structure of compound **15**.

Data for compound **15**: pale yellow powder, m. p. 179–181°C; ESI-MS: Calcd for C_27_H_30_O_16_ ([M + H]^+^), 611.19; ^1^H NMR (400 MHz, DMSO-*d*_6_) *δ*: 12.56 (s, 1H, H-5), 10.93 (s, 1H,H-4′), 8.16–8.08 (m, 2H, H-2′, 6′), 7.20–7.12 (m, 2H, H-3′, 5′), 6.47 (d, *J* = 2.1 Hz, 1H, H-8), 6.23 (d, *J* = 2.1 Hz, 1H, H-6), 5.47 (d, *J* = 7.4 Hz, 1H, 1″), 5.33 (dd, *J* = 14.0, 4.7 Hz, 2H), 5.12–5.00 (m, 4H), 4.93 (d, *J* = 4.2 Hz, 1H), 4.57 (t, *J* = 5.7 Hz, 1H), 4.29 (t, *J* = 5.6 Hz, 1H), 3.71–3.62 (m, 1H), 3.58 (dd, *J* = 11.3, 5.4 Hz, 1H), 3.49–3.39 (m, 1H), 3.40–3.37 (m, 1H), 3.32–3.15 (m, 6H), 3.09–3.01 (m, 2H, H-6″); ^13^C NMR (100 MHz, DMSO-*d*_6_) *δ*: 177.54 (C-4), 164.27 (C-7), 161.22 (C-5), 159.23 (C-4′), 156.45 (C-9), 155.60 (C-2), 133.73 (C-3), 130.57 (C-2′, 6′), 123.70 (C-1′), 115.81 (C-3′, 5′), 104.11 (C-10), 100.85 (C-1″), 100.00 (C-1‴), 98.76 (C-6), 93.73 (C-8), 77.56 (C-5″), 77.08 (C-5‴), 76.52 (C-3″), 76.43 (C-3‴), 74.18 (C-2″), 73.21 (C-2‴), 69.92 (C-4″), 69.61 (C-4‴), 60.87 (C-6″), and 60.63 (C-6‴). According to the abovementioned data and literature (Shan et al., 2020), compound **15** was identified as Kaempferol-3,7-*O*-diglucoside and its molecular formula is shown in Figure 11. MS diagrams and ^1^H-NMR and ^13^C-NMR spectra were shown in Supplementary Figures S43–S45, respectively.

Data for compound **16**: pale yellow powder, m. p. 166–168°C; ESI-MS: Calcd for C_27_H_30_O_15_([M + H]^+^), 595.15; ^1^H NMR (400 MHz, DMSO-*d*_6_) *δ*:12.56 (s, 1H, H-5), 10.86 (s, 1H, H-4′), 10.12 (s, 1H, H-7), 8.03–7.93 (m, 2H, H-2′, 6′), 6.92–6.84 (m, 2H, H-3′, 5′), 6.42 (d, *J* = 2.1 Hz, 1H, H-8), 6.21 (d, *J* = 2.1 Hz, 1H, H-6), 5.32 (d, *J* = 7.4 Hz, 1H, 1″), 4.38 (d, *J* = 1.6 Hz, 1H, H-1‴), 4.03 (s, 2H), 3.75–3.64 (m, 1H), 3.42 (dd, *J* = 3.4, 1.6 Hz, 1H), 3.32–3.25 (m, 4H), 3.69 (d, *J* = 9.8 Hz, 1H), 3.16–3.01 (m, 2H, H-6″), 0.99 (d, *J* = 6.2 Hz, 3H, H-6‴); ^13^C NMR (100 MHz, DMSO-*d*_6_) *δ*: 177.38 (C-4), 164.11 (C-7), 161.18 (C-5), 159.88 (C-4′), 156.82 (C-2), 156.48 (C-9), 133.22 (C-3), 130.84 (C-2′, 6′), 120.87 (C-1′), 115.08 (C-3′, 5′), 103.98 (C-10), 101.33 (C-1″), 100.74 (C-1‴), 98.70 (C-6), 93.72 (C-8), 76.37 (C-3″),75.75 (C-5″), 74.16 (C-2″), 71.82 (C-4‴), 70.60 (C-3‴), 70.33 (C-2‴), 69.93 (C-4″), 68.22 (C-5‴), 66.88 (C-6″), and 17.69 (C-6‴). According to the abovementioned data and literature (Dong et al., 2015), compound **16** was identified as Kaempferol-3-*O*-rutinoside and its molecular formula is shown in Figure 3. MS diagrams and ^1^H-NMR and ^13^C-NMR spectra were shown in Supplementary Figures S46–S48, respectively.

### 3.2 Disscusion

As a plant found in the desert of the Inner Mongolia, Ningxia, Gansu Region, *A. mongolicum* belongs to the genus *Allium* of the Liliaceae family (Wang et al., 2013; Wang et al., 2019). *A. mongolicum* was taken as a vegetable with local characteristics for cuisine and seasoning due to its unique flavor and high nutritional value, which can improve the cooking quality of mutton (Dong et al., 2020). There are a few reports about the extract of *A. mongolicum* to control some foodborne microorganism pathogens. The ethanol extract and aqueous extract of *A. mongolicum* showed antimicrobial activity against *Staphylococcus aureus*, *Escherichia coli, Saccharomyces cerevisiae,* and so on (Li and Luo, 2008; Liang et al., 2014). There have been some reports that essential oil, polysaccharides, and flavonoids of *A. mongolicum* exhibited good inhibitory activity against foodborne microorganism pathogens such as *Staphylococcus aureus*, *Escherichia coli,* and *Salmonella enteritidi* (Wu, et al., 2011; Sa, et al., 2014; Muqier et al., 2017). In this study, the effects of different extracts of *A. mongolicum* on agricultural pathogenic fungi were studied. It was found that methanol extract and petroleum ether extract of *A. mongolicum* showed a certain inhibitory effect on tested pathogenic fungi such as *Fusarium oxysporum* and *Botrytis cinerea*. Therefore, the extract of *A. mongolicum* has potential value as a botanical fungicide for the further study.

The chemical composition of *A. mongolicum* is diverse. So far, different chemical compositions of extracts of *A. mongolicum* have been discovered based on the reference and the related reports. Thirty-one flavonoids and phenolic acids were obtained and identified in the study of the effect of *A. mongolicum* to improve gastrointestinal function (Dong et al., 2020). The essential oils of *A. mongolicum* were analyzed and identified by gas chromatography-mass spectrometry and NIST Ms Search 2.0 database to identify 37 compounds, most of which were sulfur compounds such as three dimethyl sulfide, diallyl disulphide, and so on (Wu, et al., 2011). In this study, the chemical composition of methanol extract of *A. mongolicum* were separated and identified by the mass spectrum, HPLC, ^1^H NMR, and ^13^C NMR spectra to gain sixteen compounds, which include nine flavonoids and six phenolic acids (cinnamic acid derivatives **1**, **2**, **3**, and **12**; caffeic acid derivatives **10** and **11**) and tryptophan. Some of the flavonoids here have been reported in *A. mongolicum* (compounds **4**, **5**, **6**, **15**, and **16**), and those compounds are secondary metabolites, which are found in most *Allium* plants such as *Allium cepa* L., *Allium fistulosum* L., and *Allium sativum* L. and exert multiple biological activities such as antioxidant and gastrointestinal motility effect improvement. Three phenolic acids (cinnamic acid derivatives **1**, **2**, and **3**) were separated in *A. mongolicum* for the first time. Cinnamic acid is organic acid occurring naturally in plants (Sova, 2012; Merlani et al., 2019). They have been reported to have antibacterial and antifungal activities and are considered promising lead compounds for structural modification to discover bioactive compounds with significant activity (Guzman, 2014; Khan et al., 2021). Based on the existing reports about the antimicrobial activity of cinnamic acid and its derivatives, therefor it is presumed that the antifungal activity of crude extract of *A. mongolicum* may be partly due to the three phenolic acids (**1**, **2**, and **3**), which antimicrobial activity is worth further studying. In subsequent experiments, we can take cinnamic acid as a lead compound to synthesize a series of cinnamic acid derivatives with similar structures reported in this article and make a systematic study on their antimicrobial activity. Among the nine isolated flavonoids, there are four new compounds (flavonoids **7**, **8**, **9**, and **13**) found in *A. mongolicum*. As is known, natural flavonoids exist in the form of its glycosides. Compared with flavonoids that have been reported, the four new compounds here are different mainly on the position and number of glucosyl of flavanone glycosides. This study laid a foundation for the further development and utilization of extracts of *A. mongolicum* as botanical fungicides.

## 4 Conclusion

In this article, based on the certain inhibitory activity against nine tested pathogens of extracts of *A. mongolicum*, 16compounds were isolated and identified by HPLC, NMR, and ESI-MS, and seven compounds including cinnamic acid derivatives and flavonoids were gained from *A. mongolicum* for the first time. According to our knowledge, the antimicrobial research of *A. mongolicum* almost focused on controlling some foodborne microorganism pathogens. Therefore, this study laid a foundation for the systematic research on the inhibitory effect of compounds of *A. mongolicum* on common plant-pathogenic fungi also provides good guidance for further study to discover potential antifungal agents. The follow-up study is in process, and more interesting results deserve attention.

## Data Availability

The original contributions presented in the study are included in the article/Supplementary Material, and further inquiries can be directed to the corresponding authors.
