# Supplementary material for: Antifungal effects and active compounds of the leaf of Allium mongolicum Regel
Source: Front Chem. 2022 Aug 24;10:993893. doi: 10.3389/fchem.2022.993893 (PMC9451007; doi:10.3389/fchem.2022.993893)
Supplement: Supplementary file 1 [file DataSheet1.PDF]

SUPPLEMENTARY MATERIAL

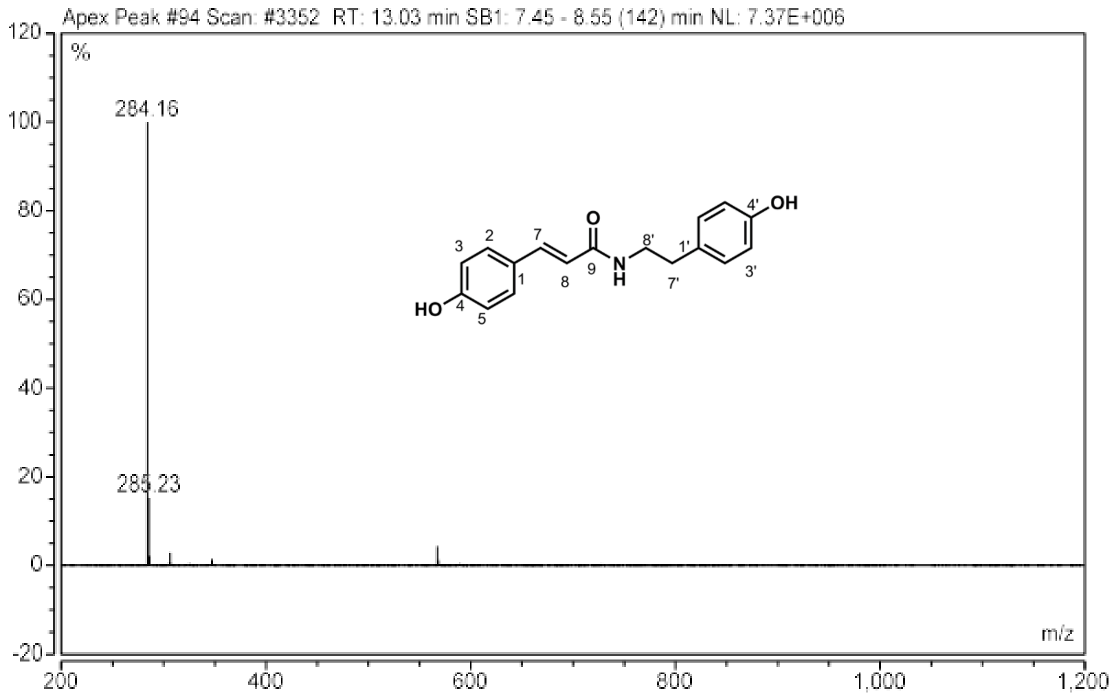

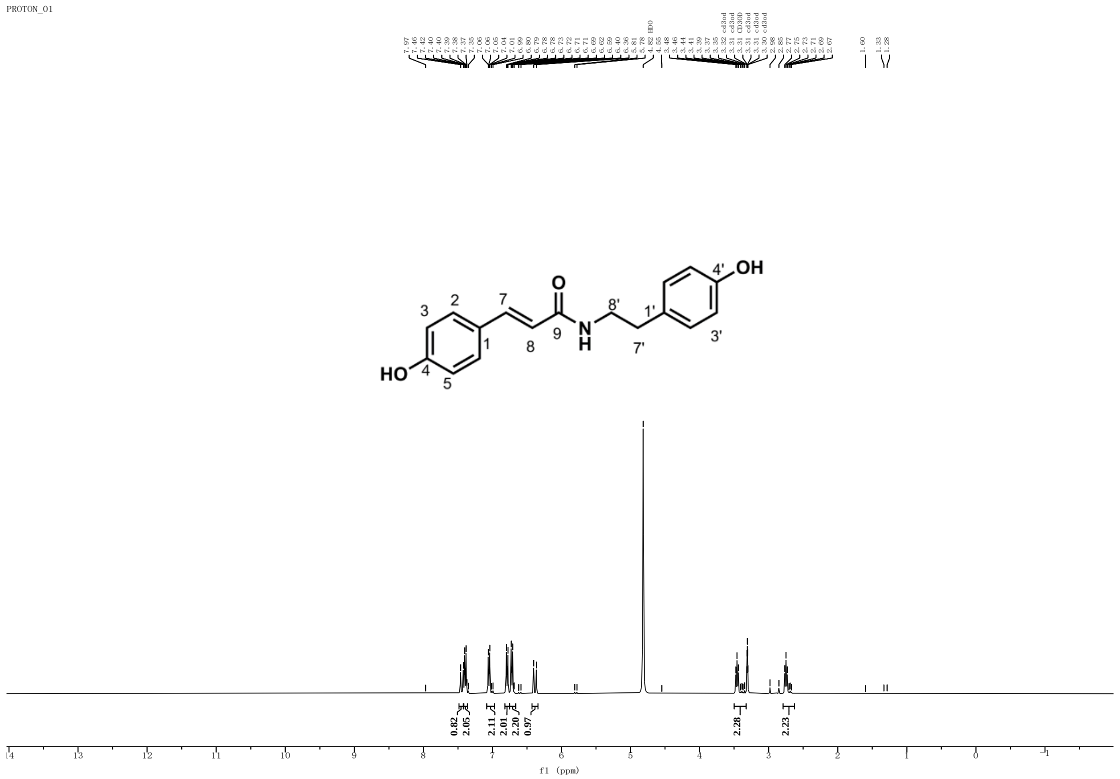

Figure S2 ^{1}H-NMR spectra of Compound **1**

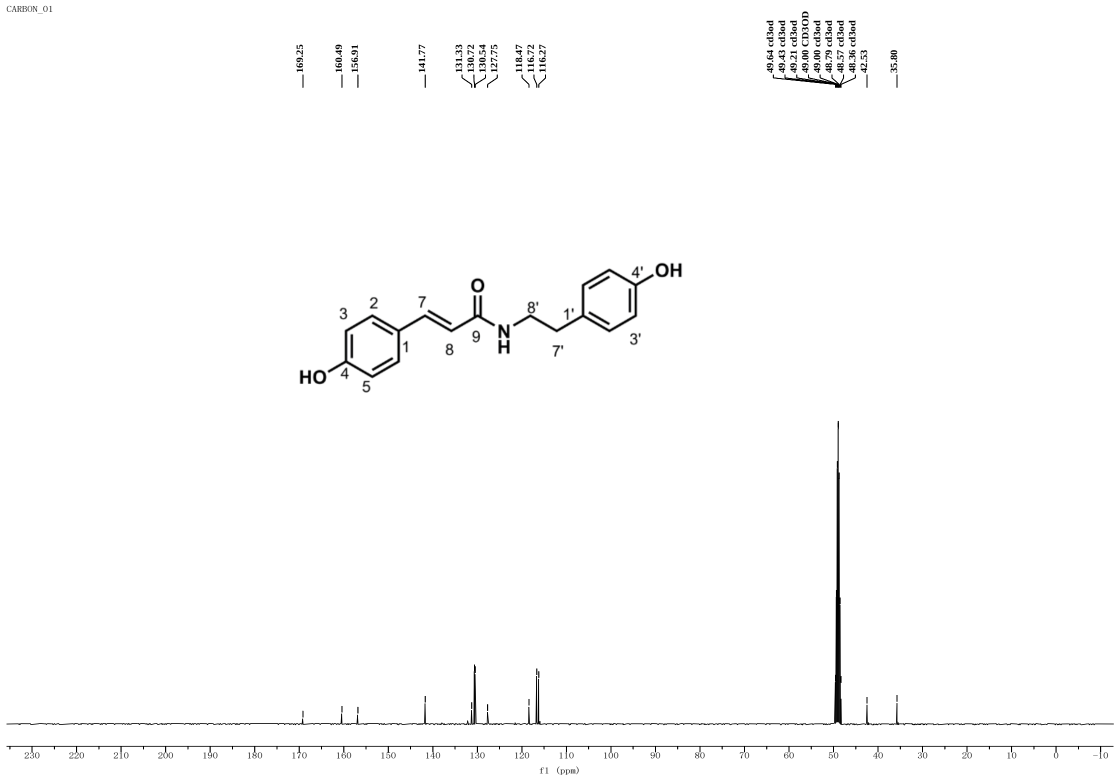

Figure S3 ^{13}C-NMR spectra of Compound **1**

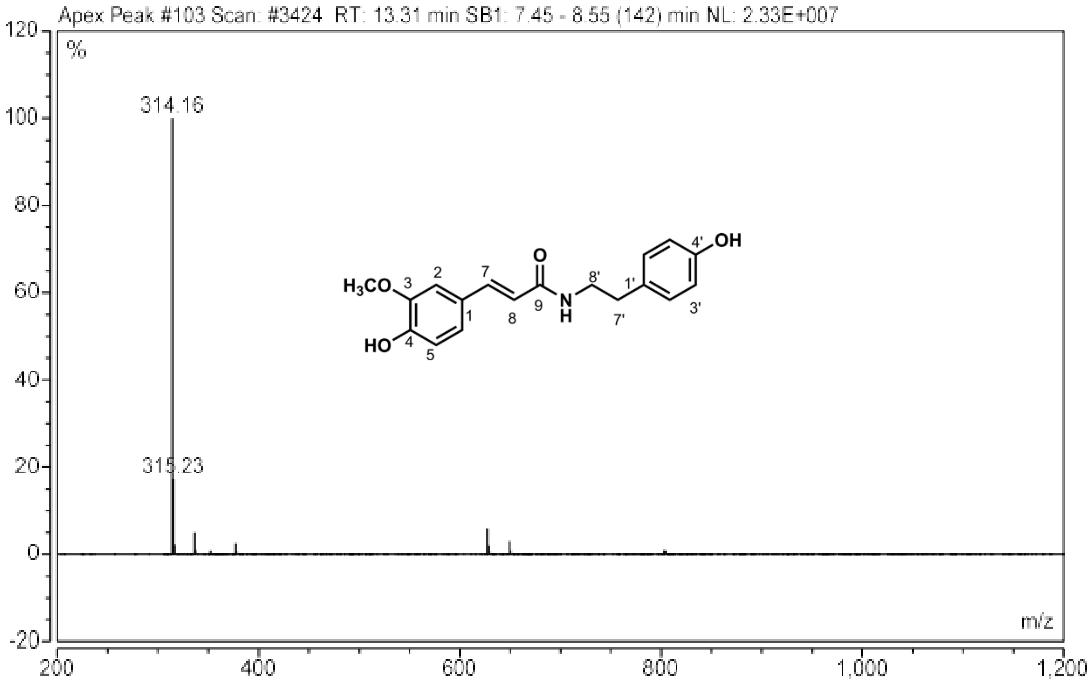

Figure S4 MS diagram of the Compound **2**

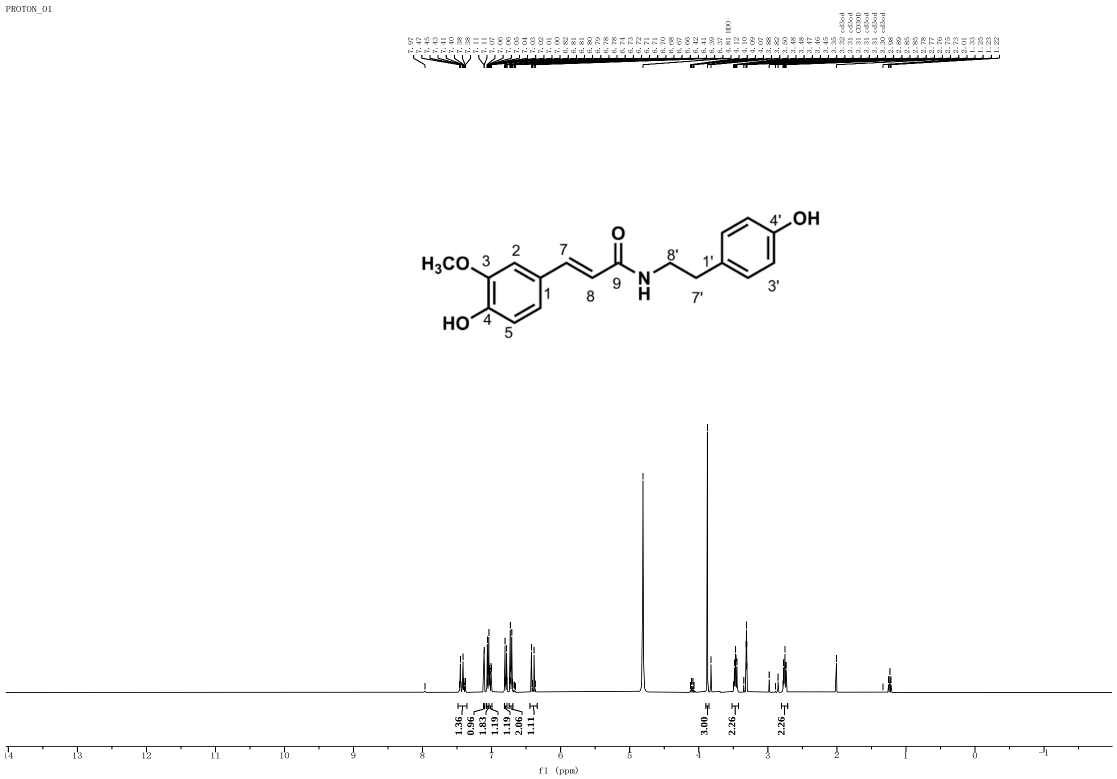

Figure S5 ^{1}H-NMR spectra of Compound **2**

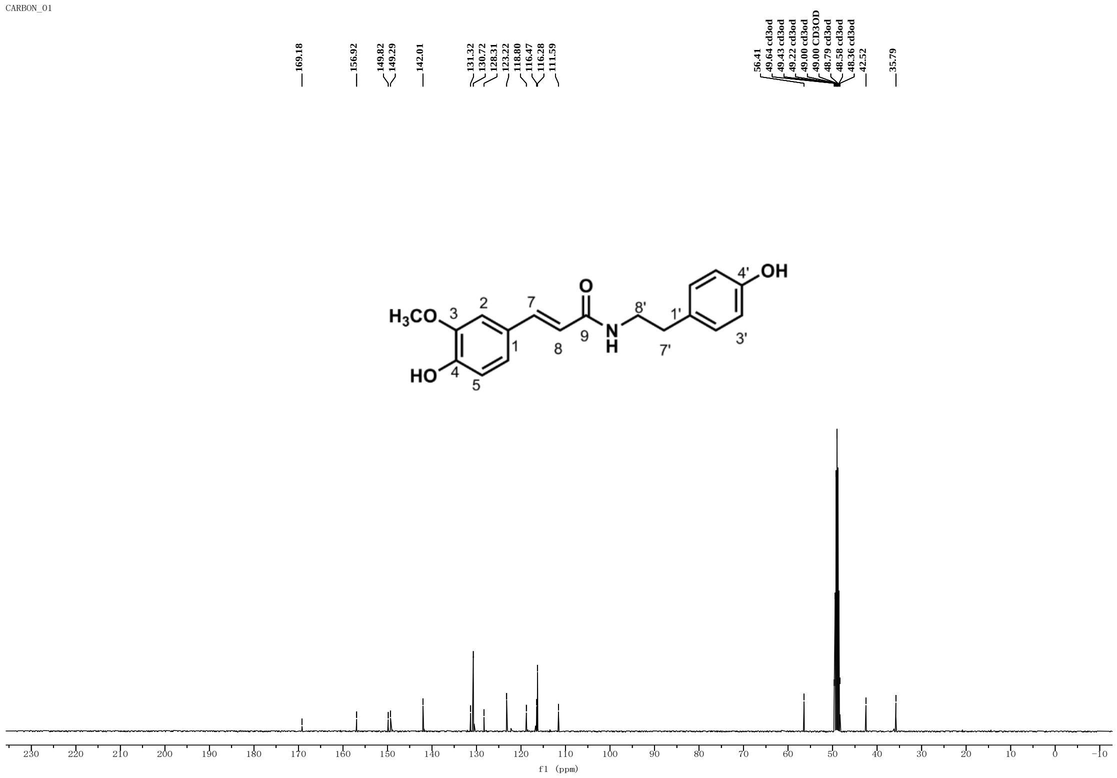

Figure S6 ^{13}C-NMR spectra of Compound **2**

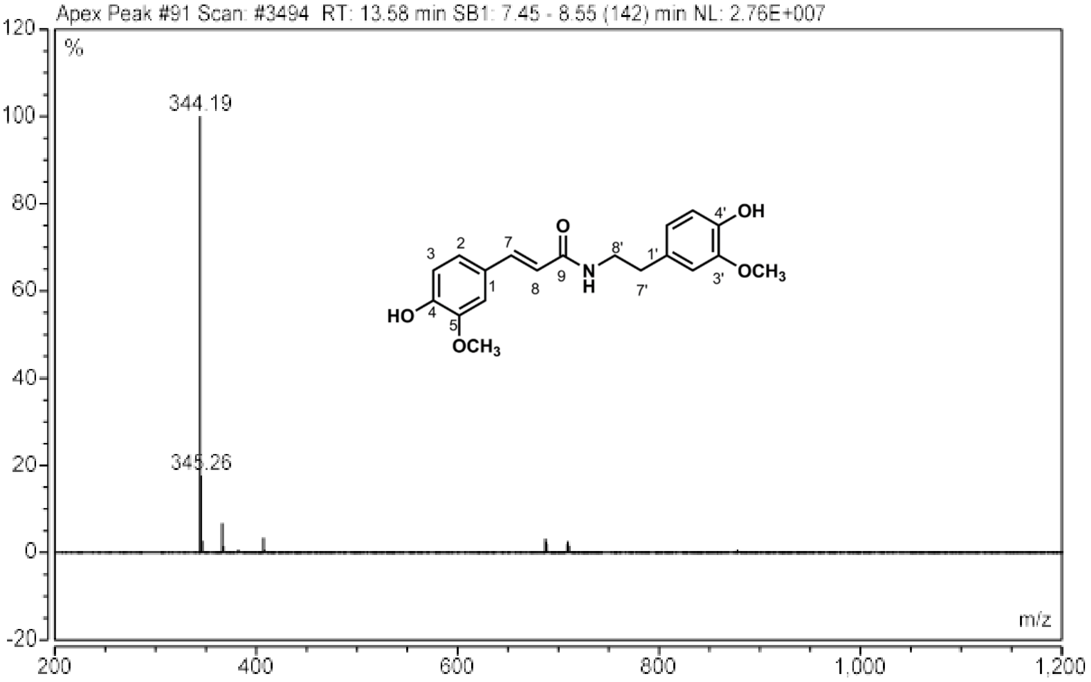

Figure S7 MS diagram of the Compound **3**

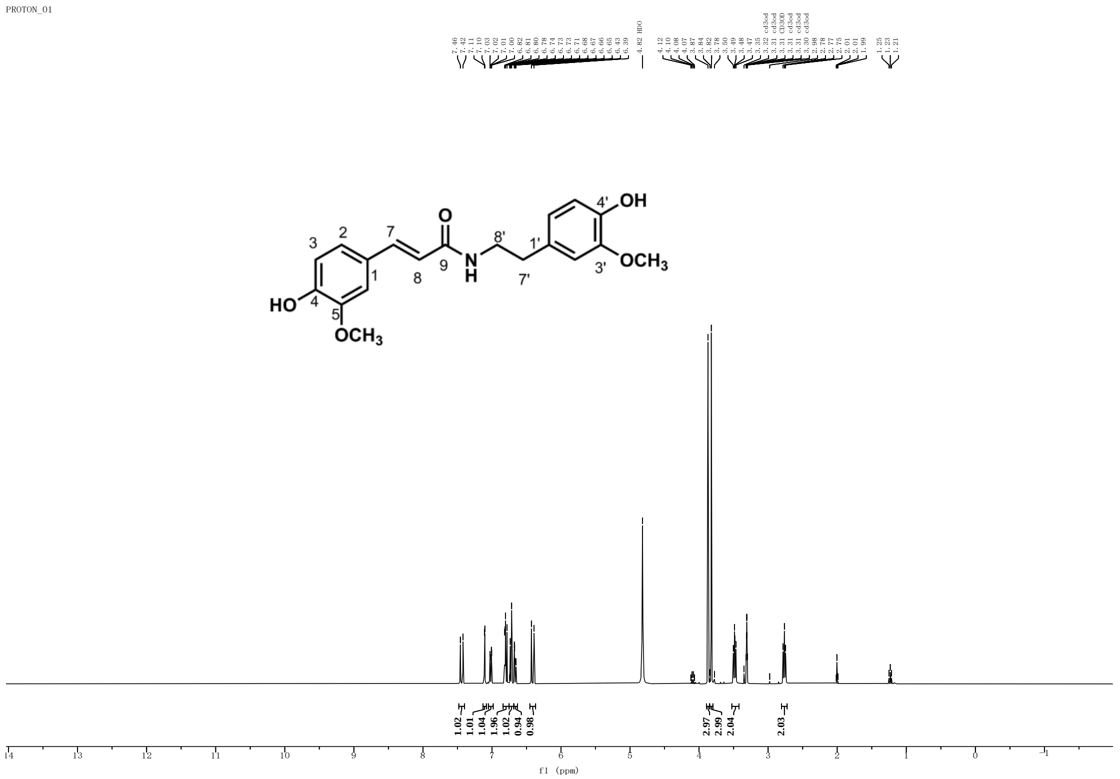

Figure S8 ^{1}H-NMR spectra of Compound **3**

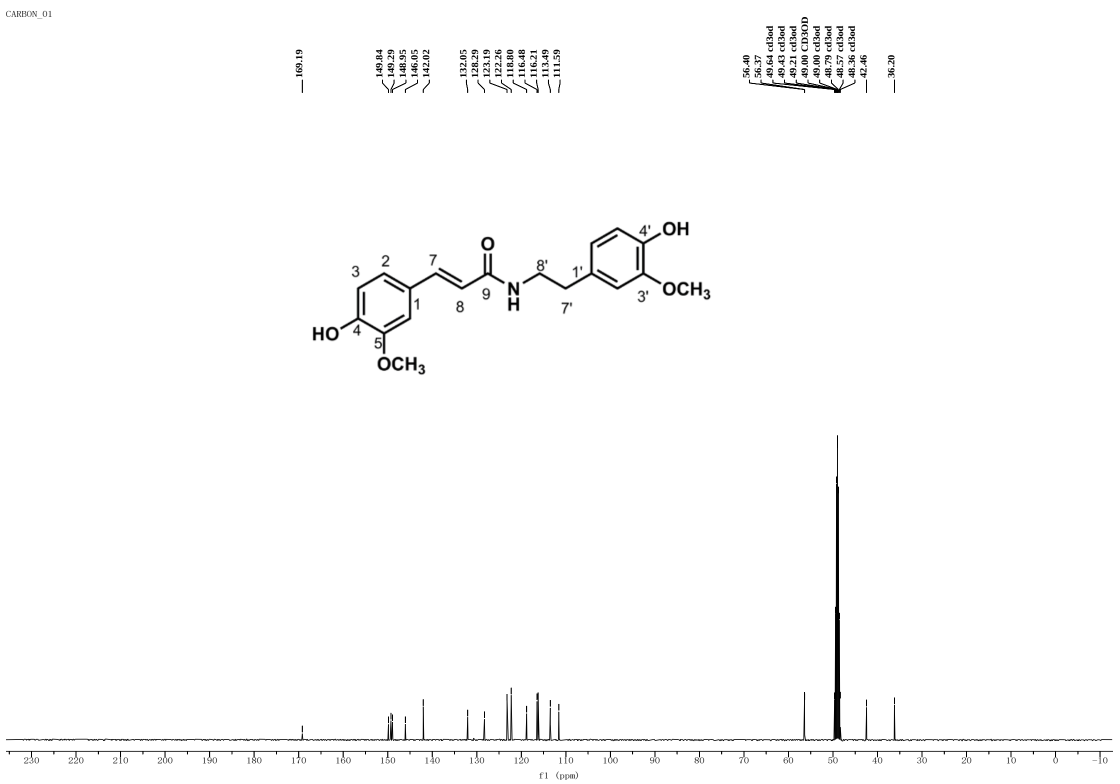

Figure S9 ^{13}C-NMR spectra of Compound **3**

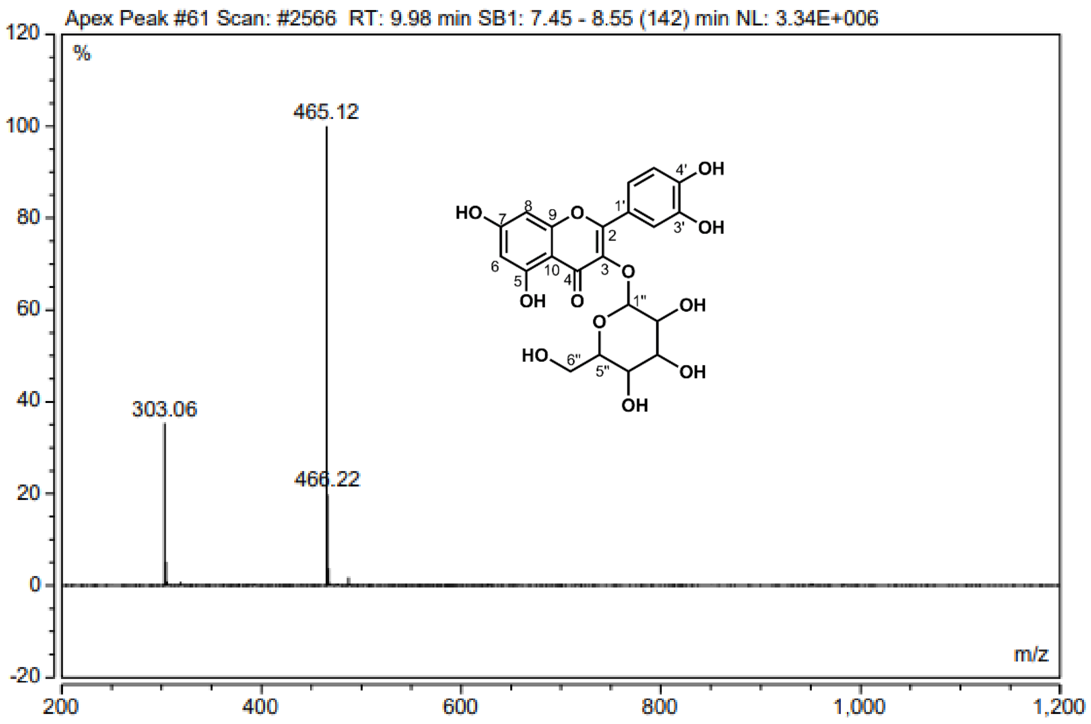

Figure S10 MS diagram of the Compound **4**

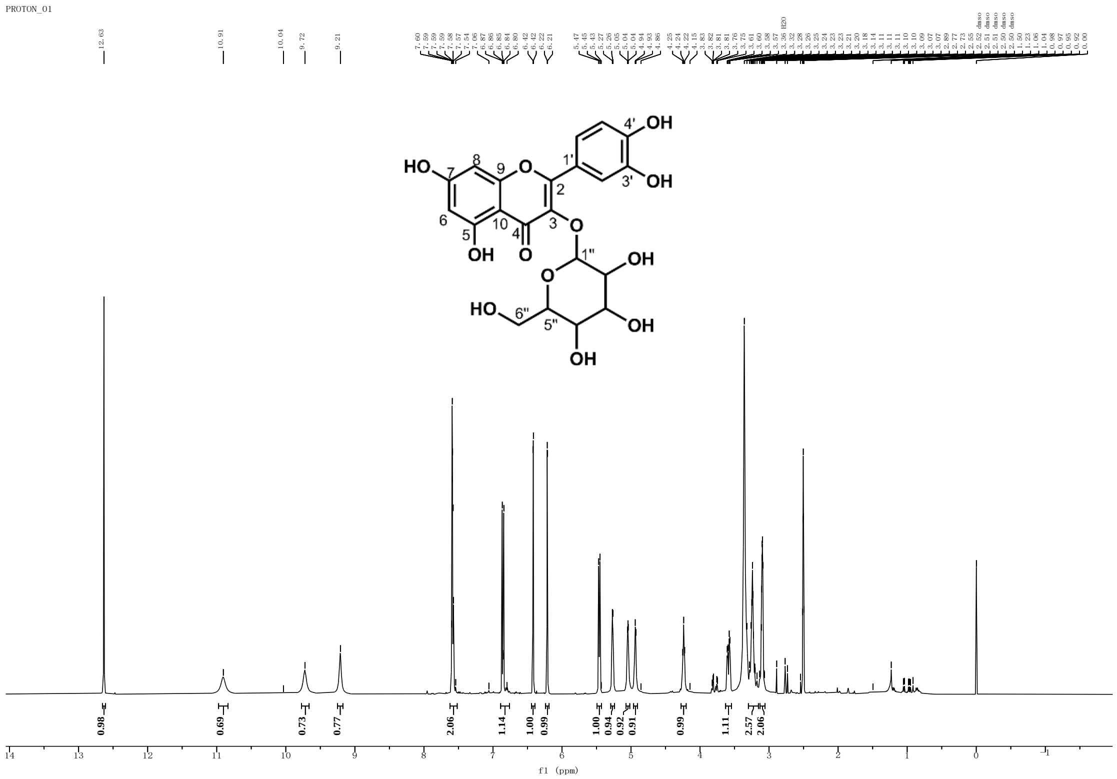

Figure S11 ^{1}H-NMR spectra of Compound **4**

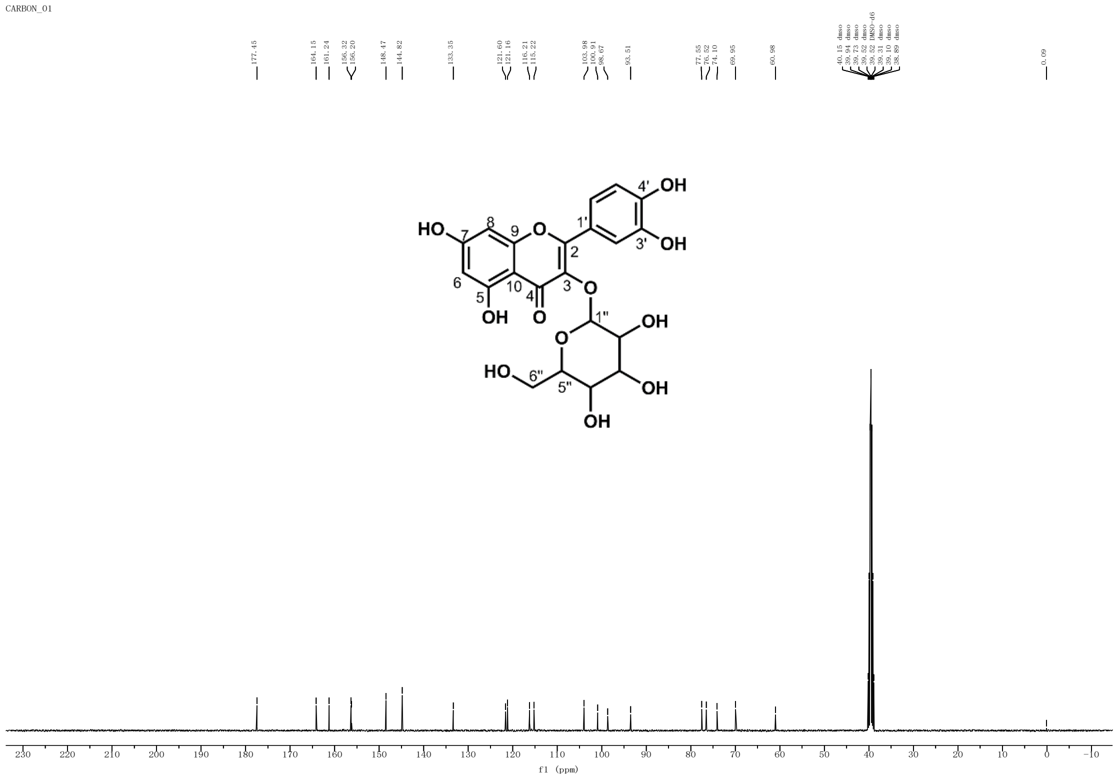

Figure S12 ^{13}C-NMR spectra of Compound **4**

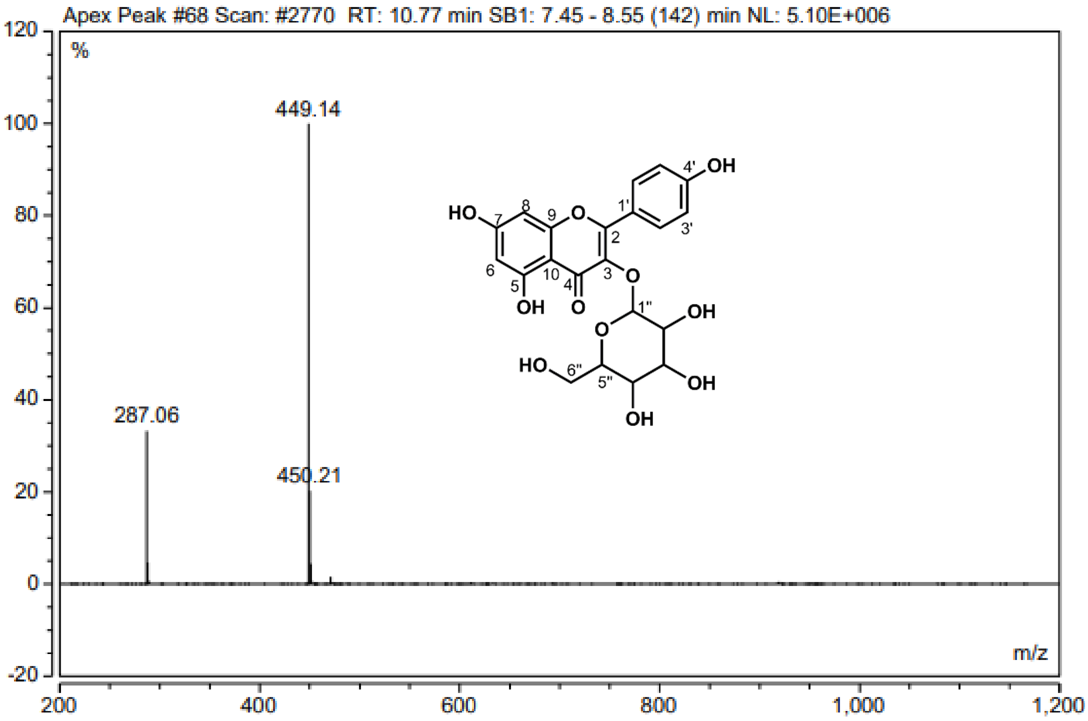

Figure S13 MS diagram of the Compound **5**

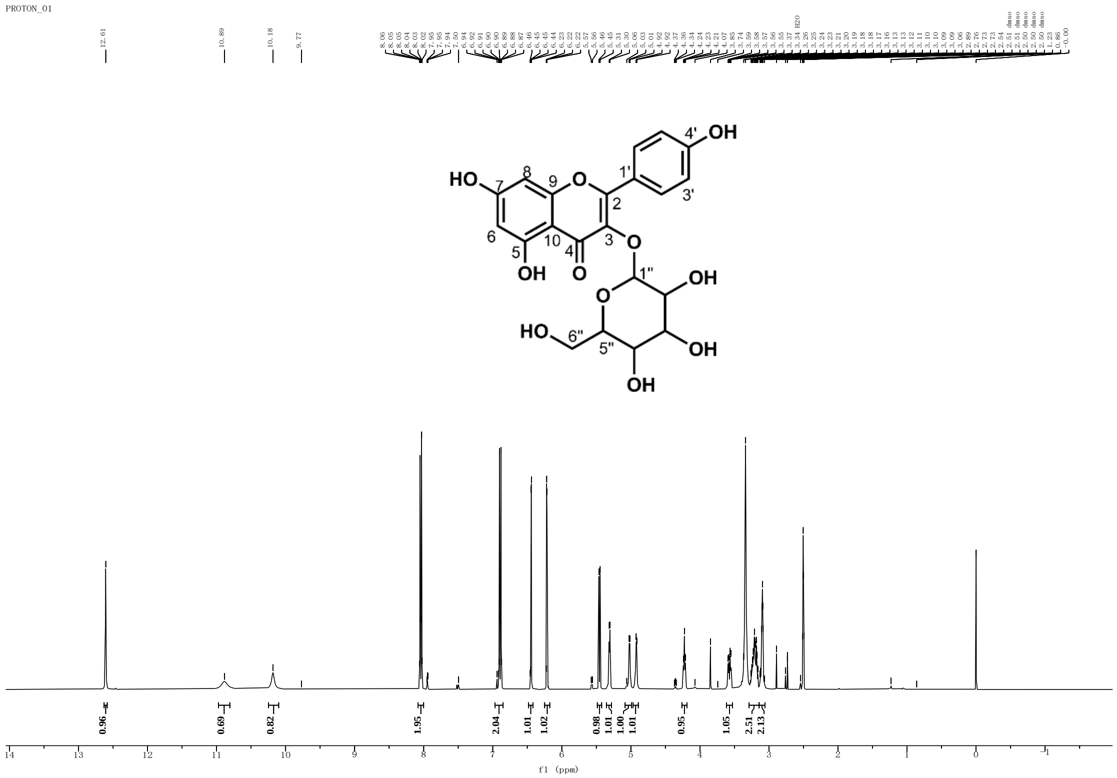

Figure S14 ^{1}H-NMR spectra of Compound **5**

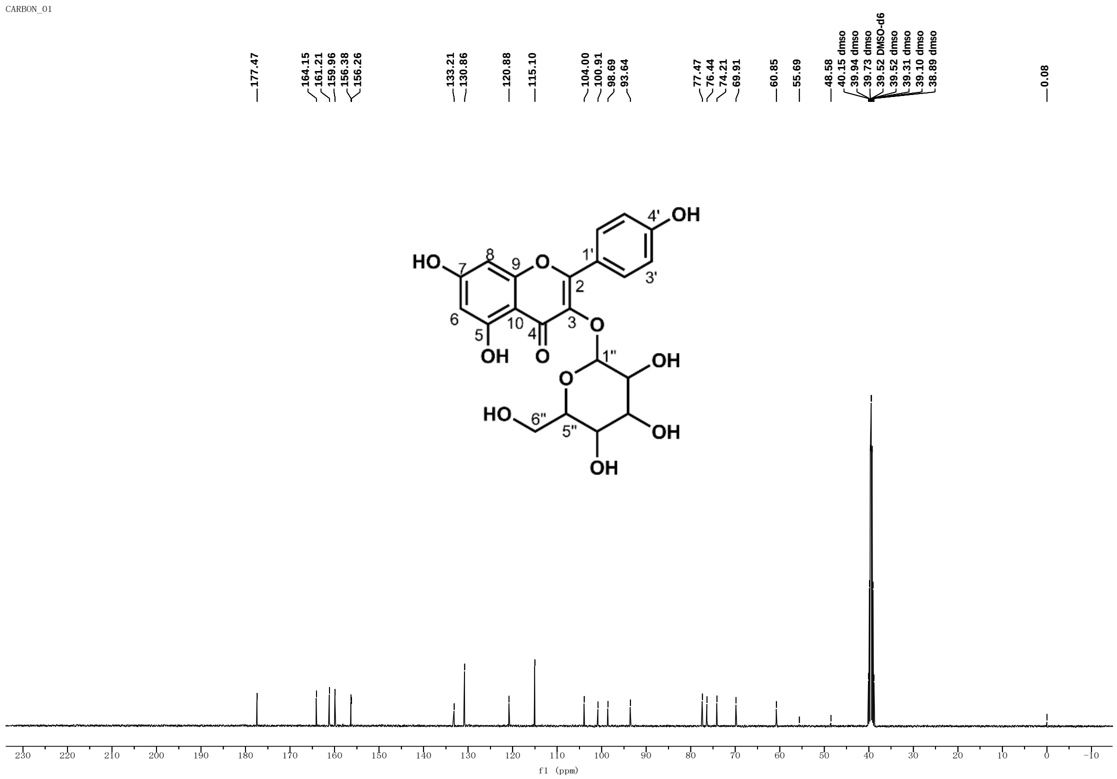

Figure S15 ^{13}C-NMR spectra of Compound **5**

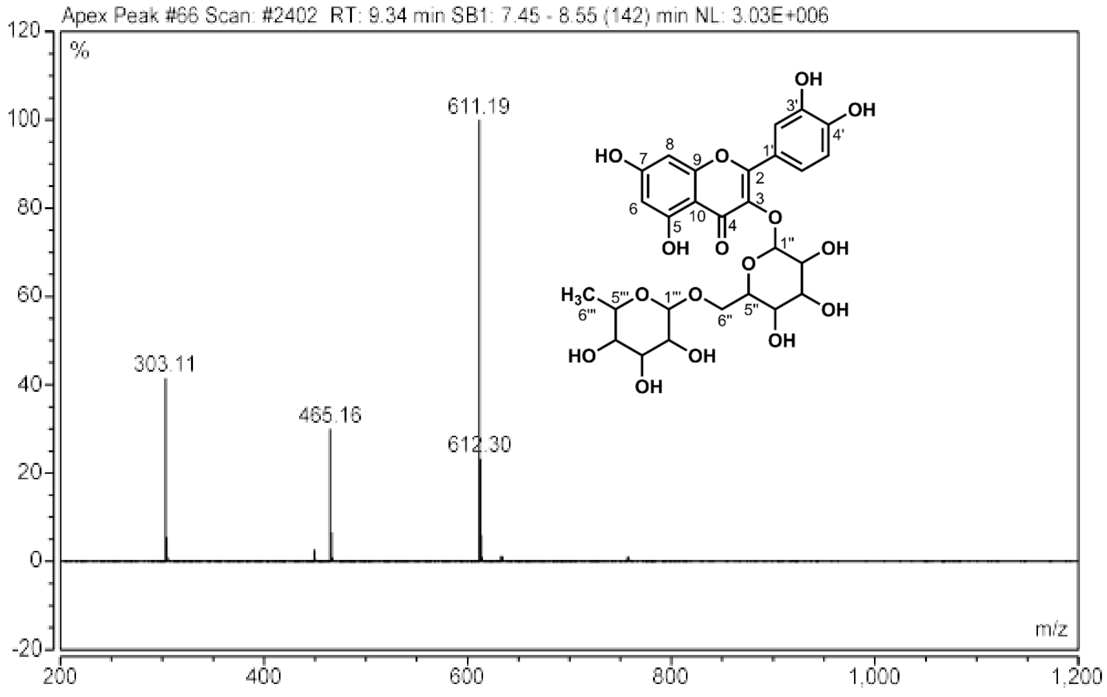

Figure S16 MS diagram of the Compound **6**

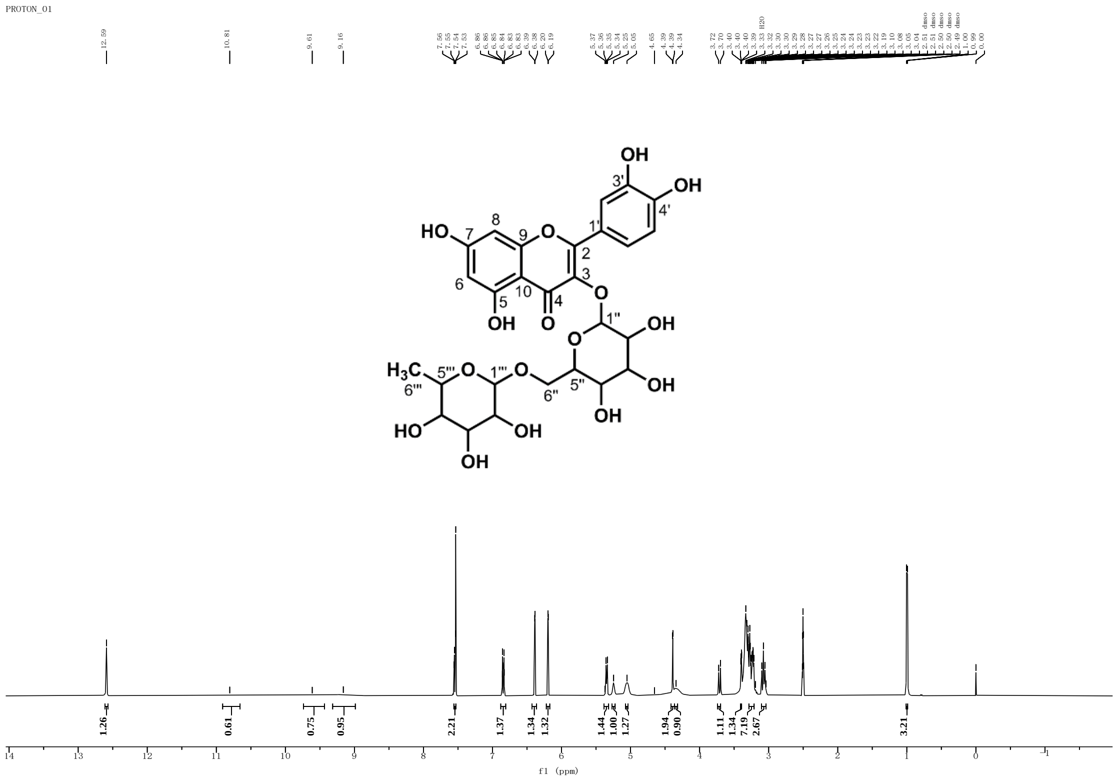

Figure S17 ^{1}H-NMR spectra of Compound **6**

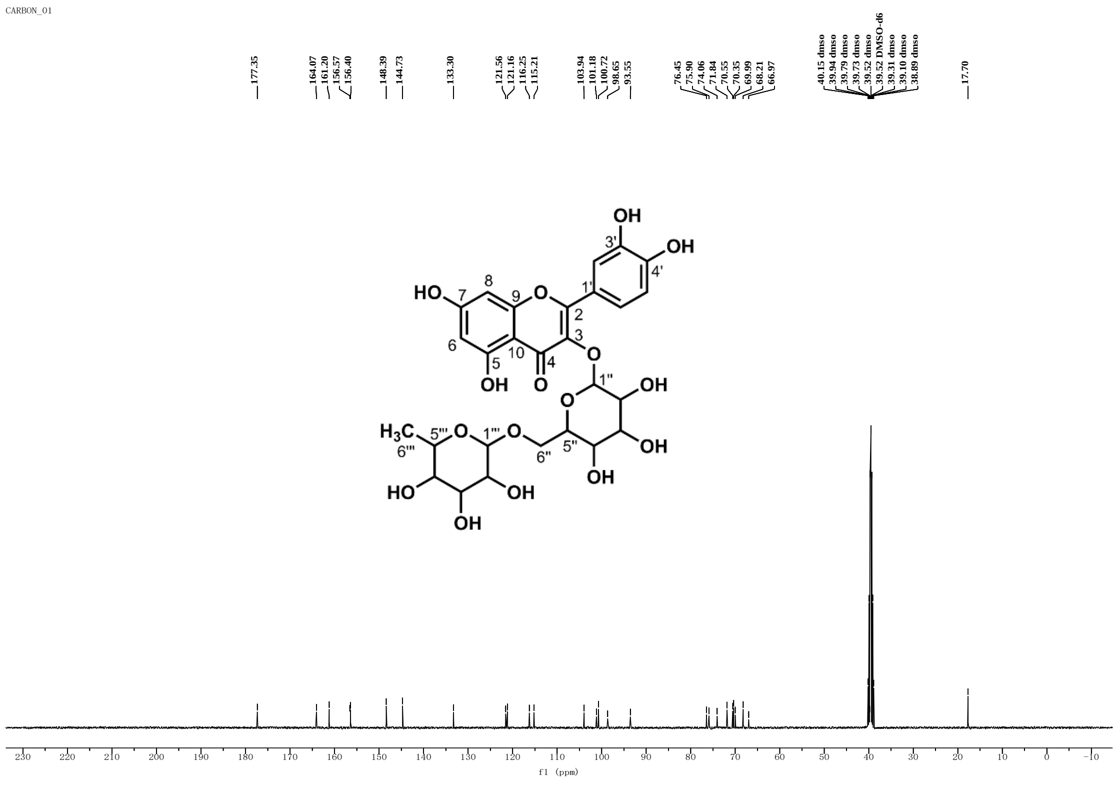

Figure S18 ^{13}C-NMR spectra of Compound **6**

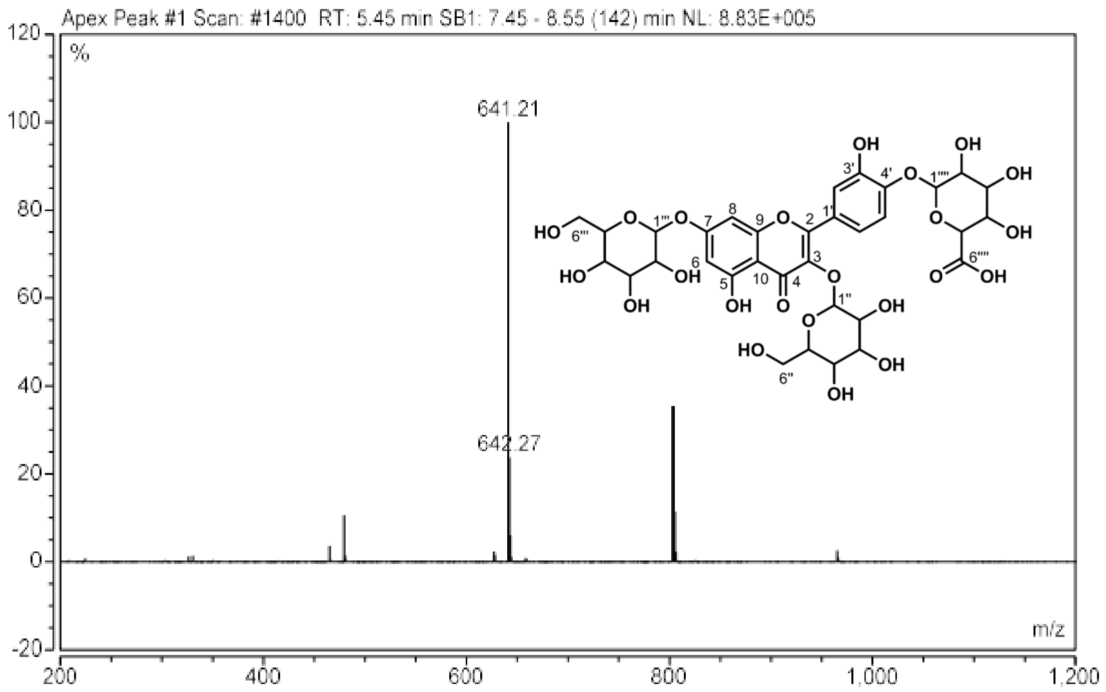

Figure S19 MS diagram of the Compound **7**

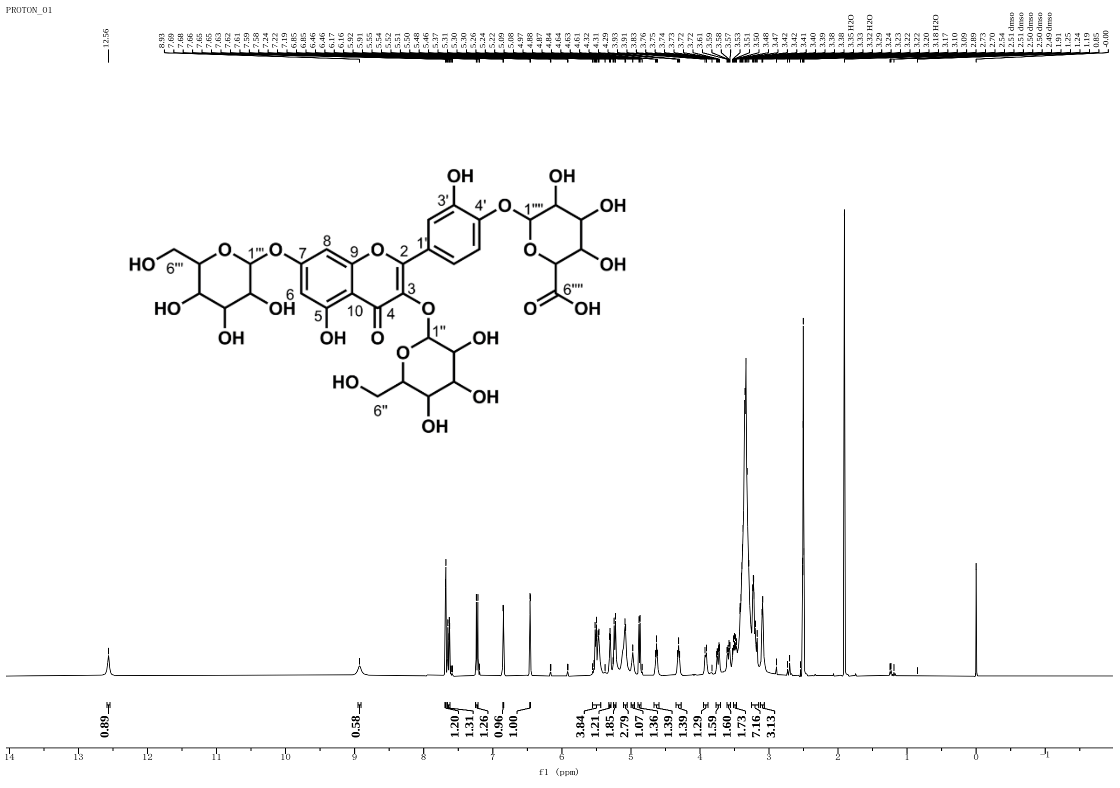

Figure S20 ^{1}H-NMR spectra of Compound **7**

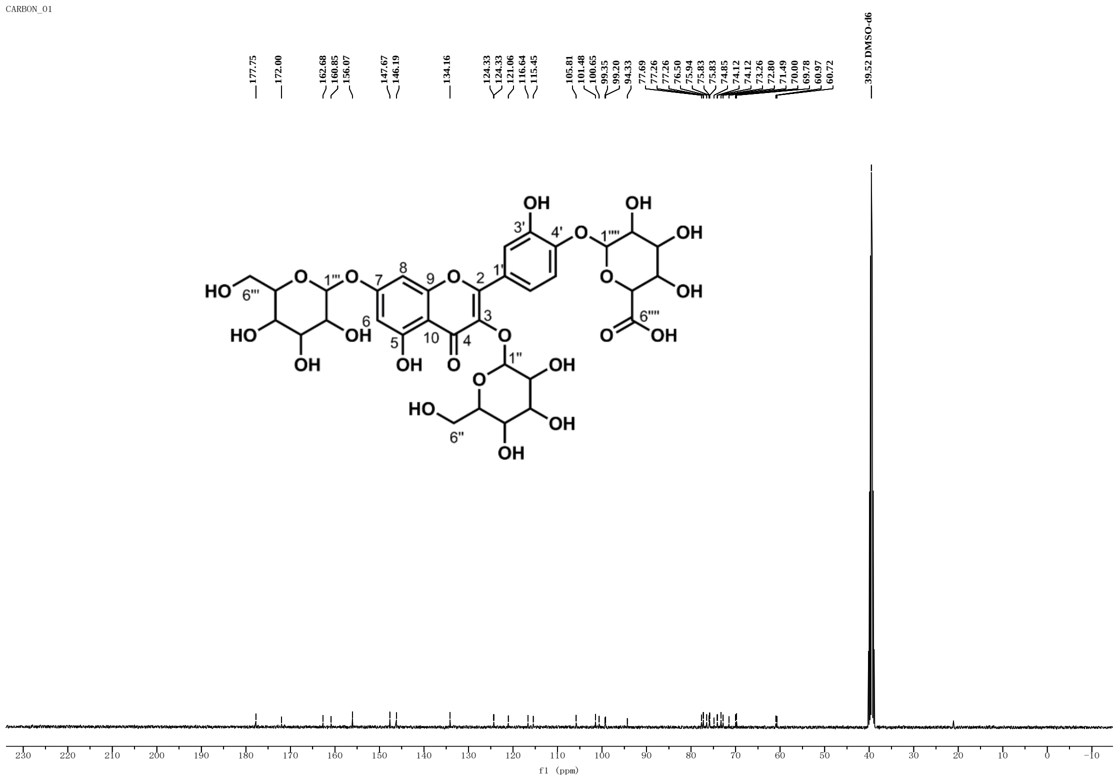

Figure S21 ^{13}C-NMR spectra of Compound **7**

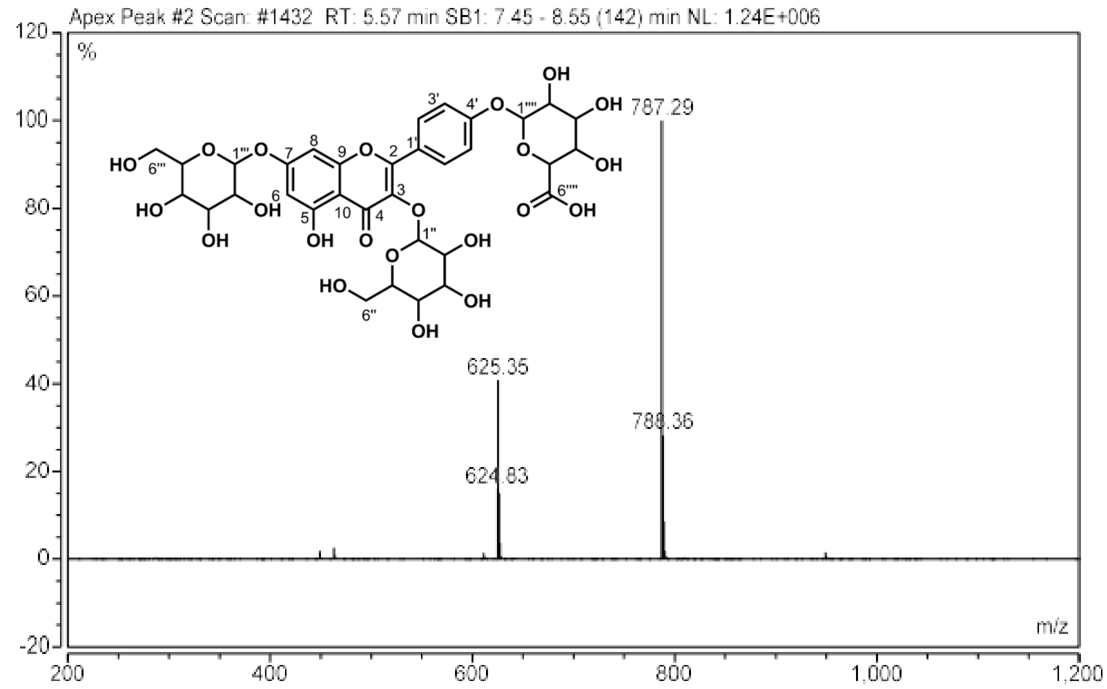

Figure S22 MS diagram of the Compound **8**

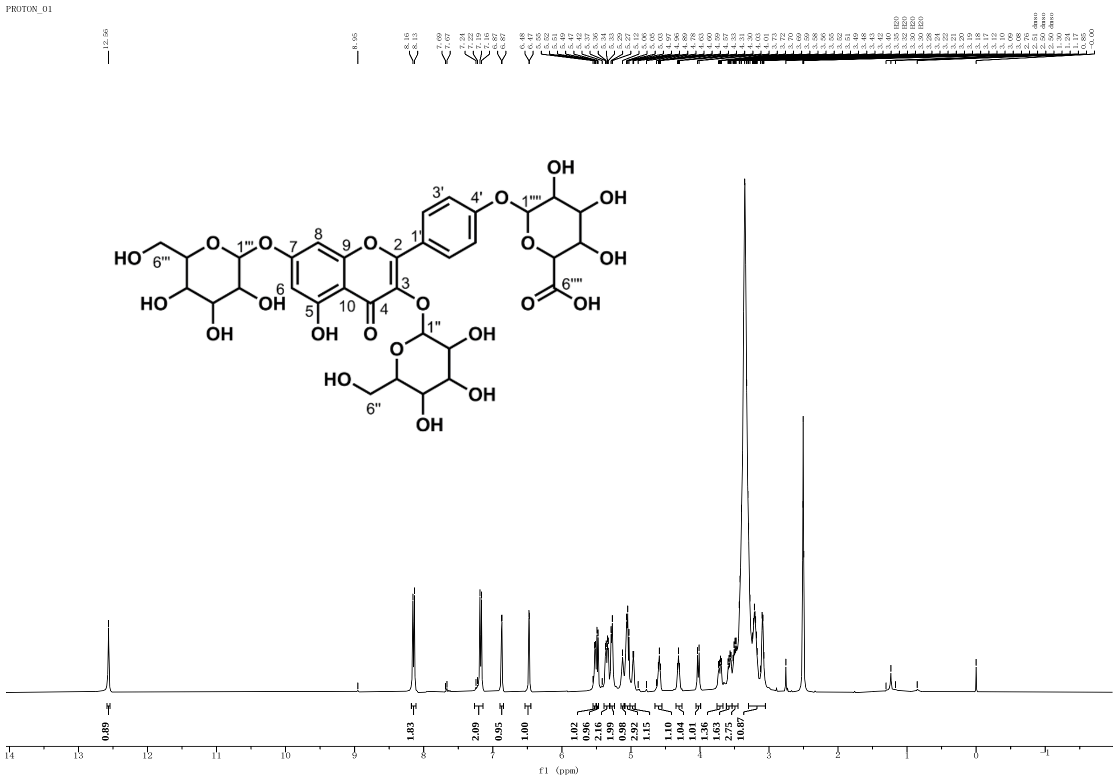

Figure S23 ^{1}H-NMR spectra of Compound **8**

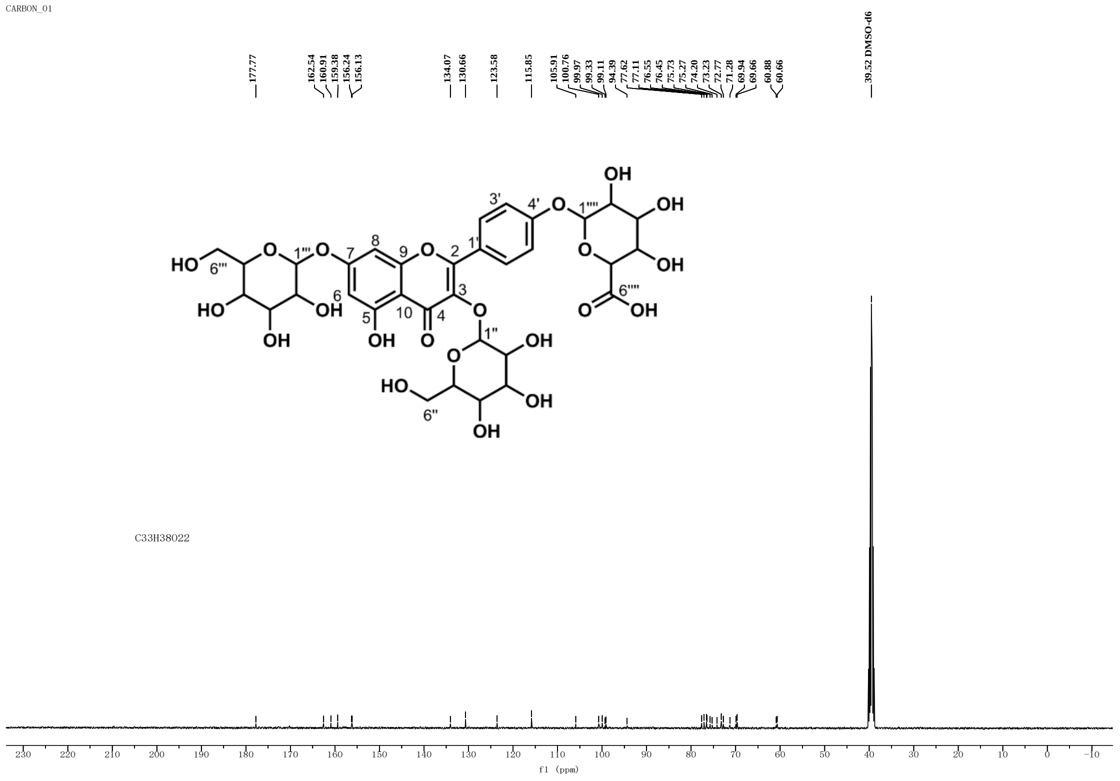

Figure S24 ^{13}C-NMR spectra of Compound **8**

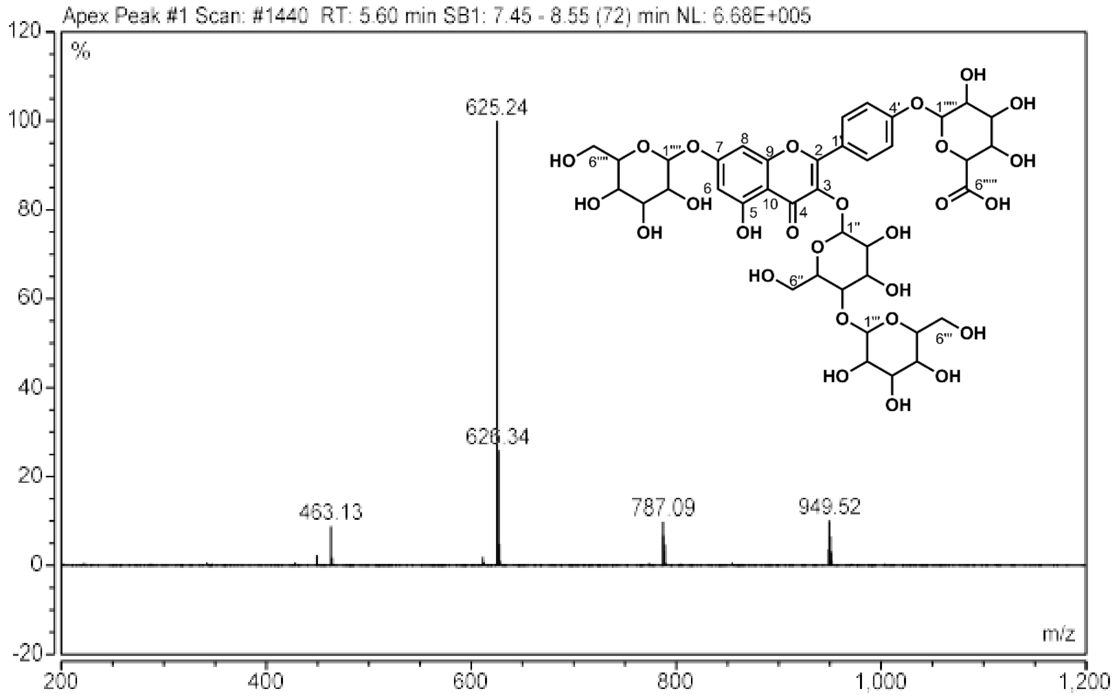

Figure S25 MS diagram of the Compound **9**

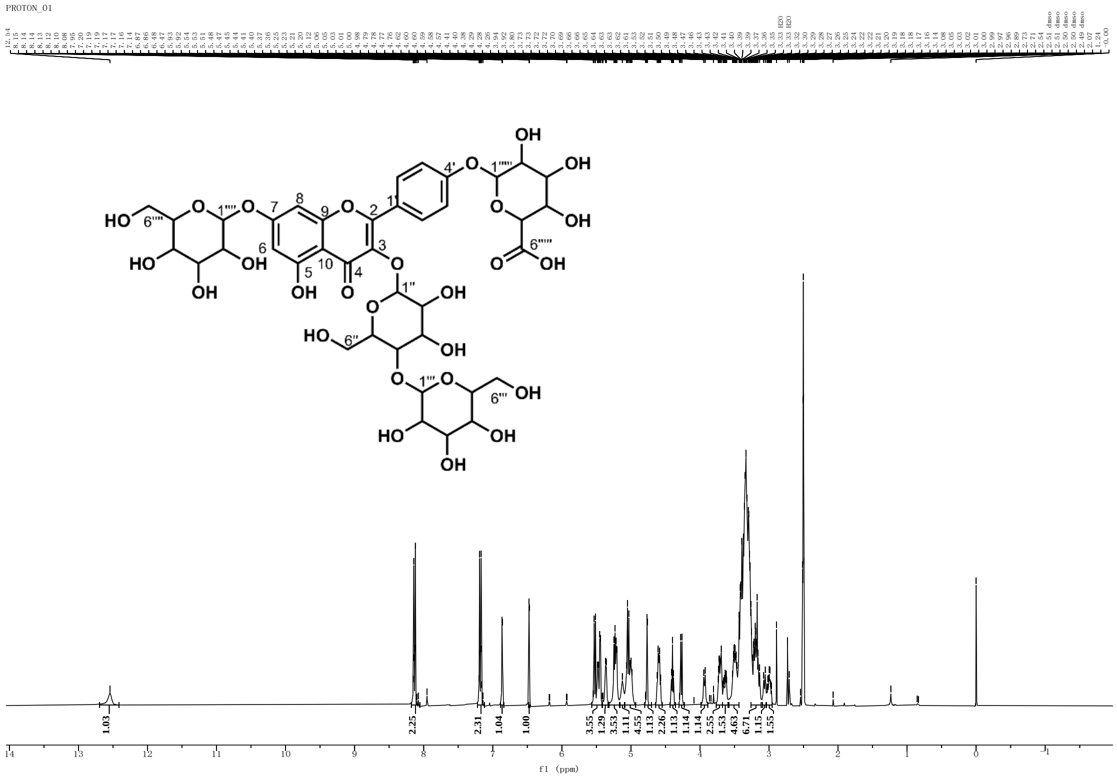

Figure S26 ^{1}H-NMR spectra of Compound **9**

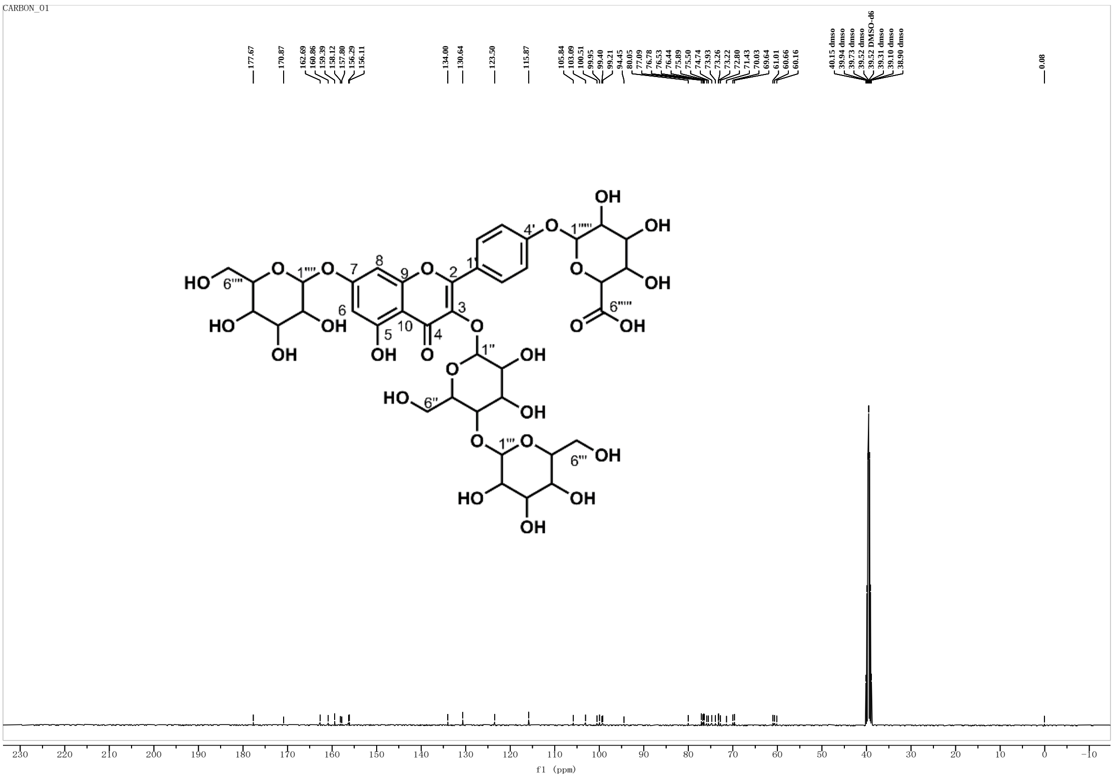

Figure S27 ^{13}C-NMR spectra of Compound **9**

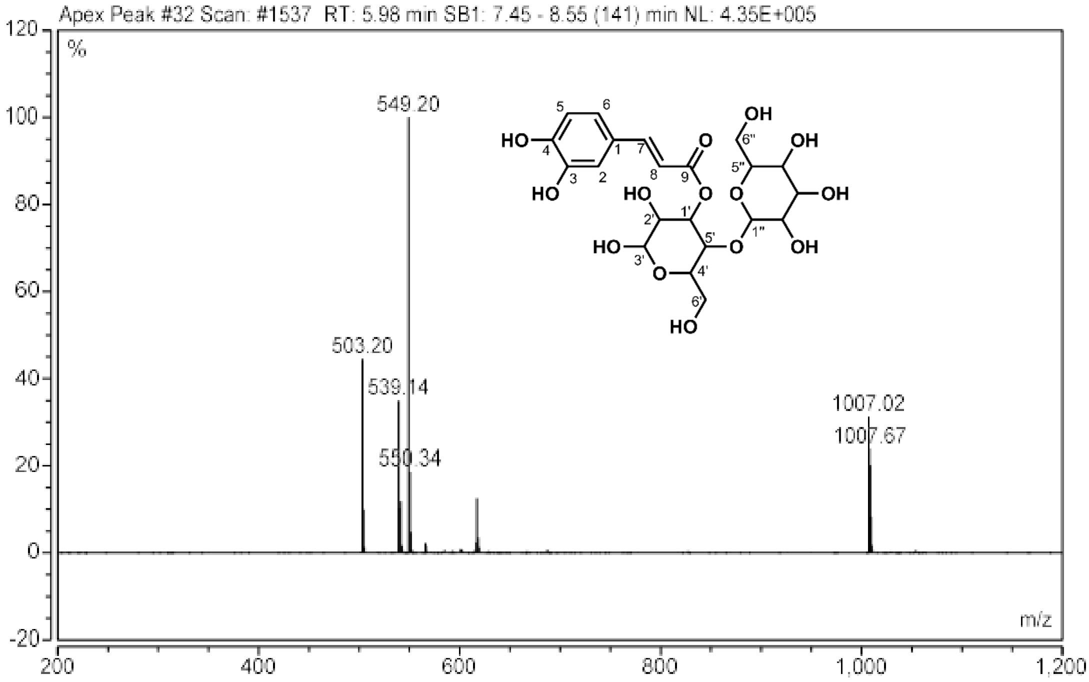

Figure S28 MS diagram of the Compound **10**

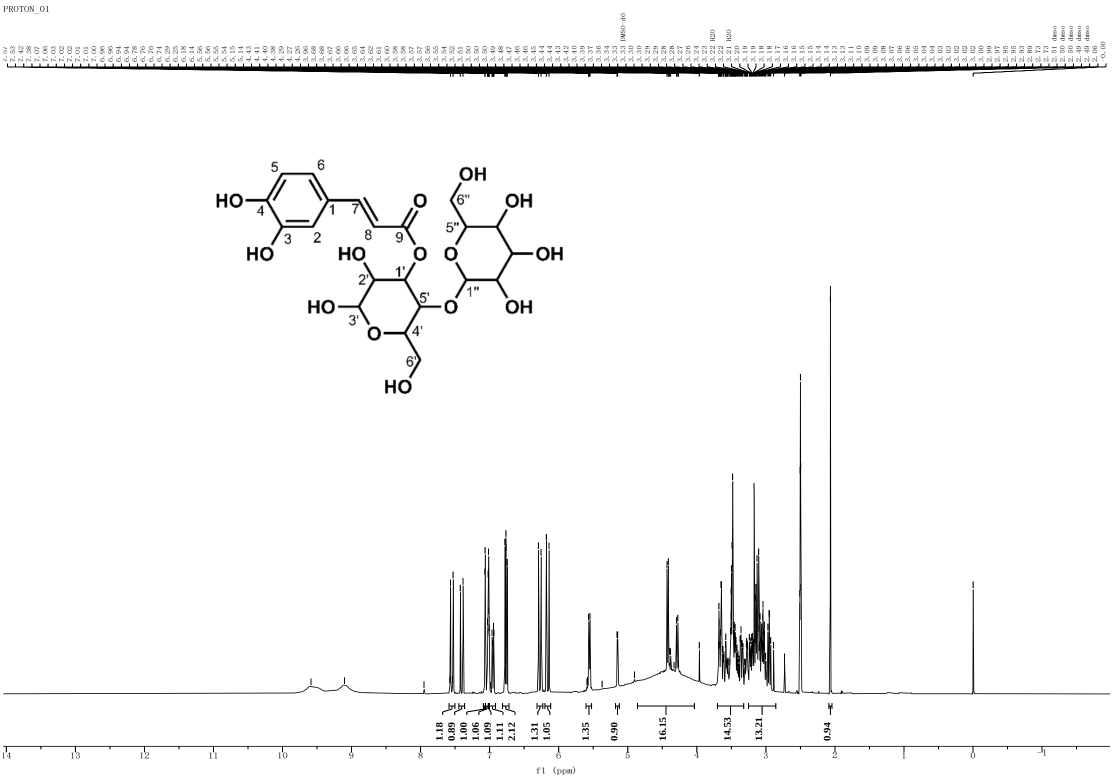

Figure S29 ^{1}H-NMR spectra of Compound **10**

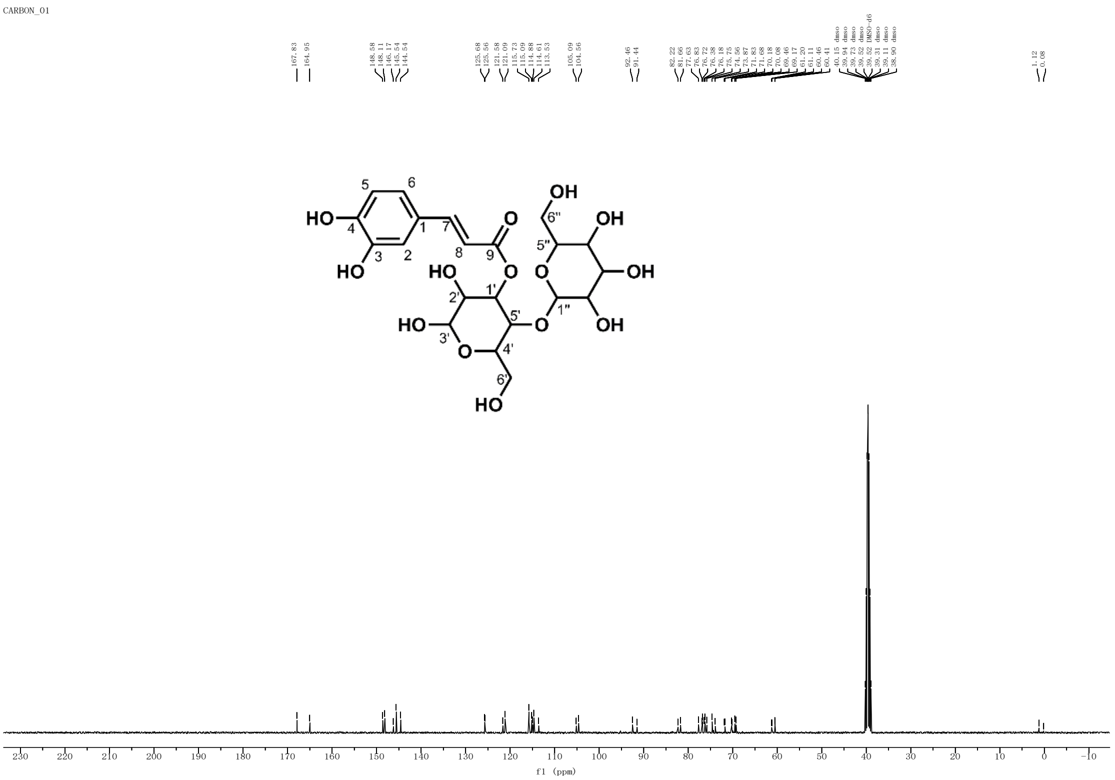

Figure S30 ^{13}C-NMR spectra of Compound **10**

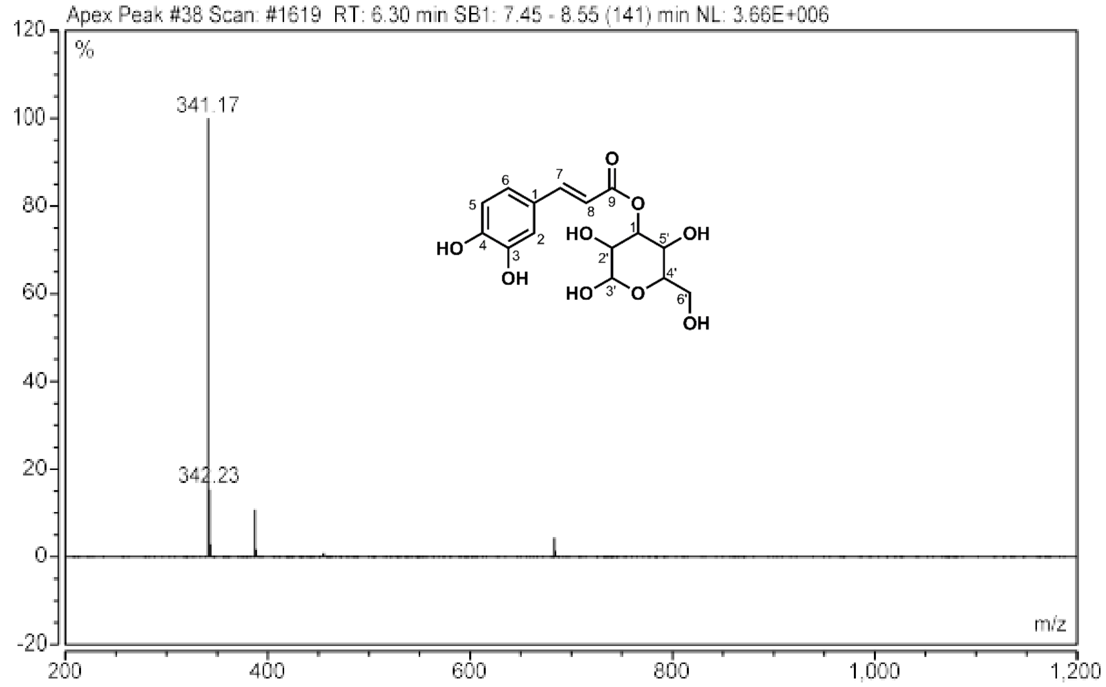

Figure S31 MS diagram of the Compound **11**

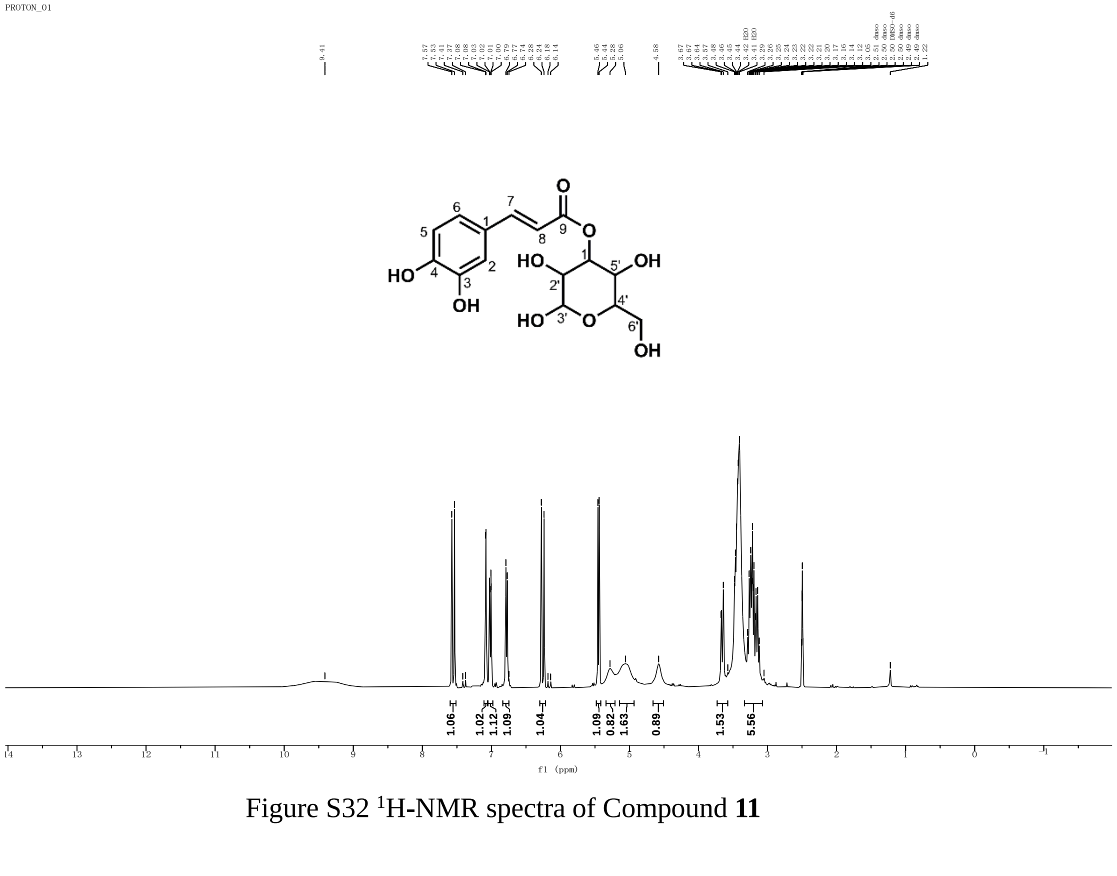

Figure S32 ^{1}H-NMR spectra of Compound **11**

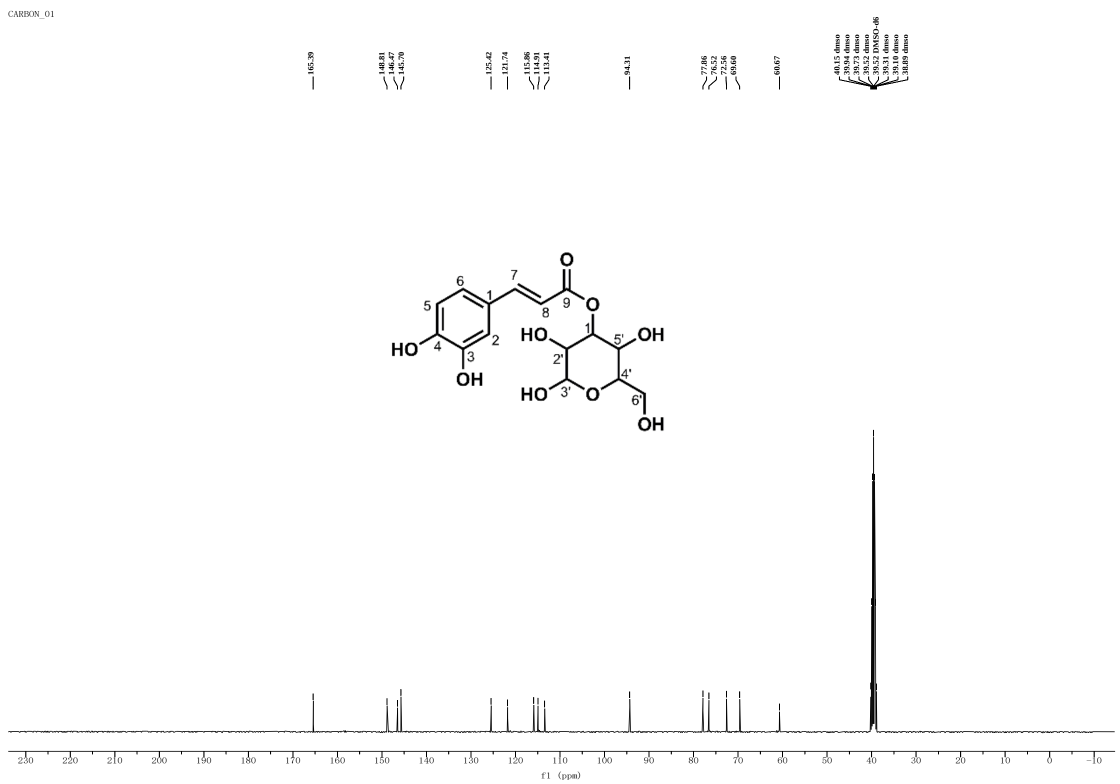

Figure S33 ^{13}C-NMR spectra of Compound **11**

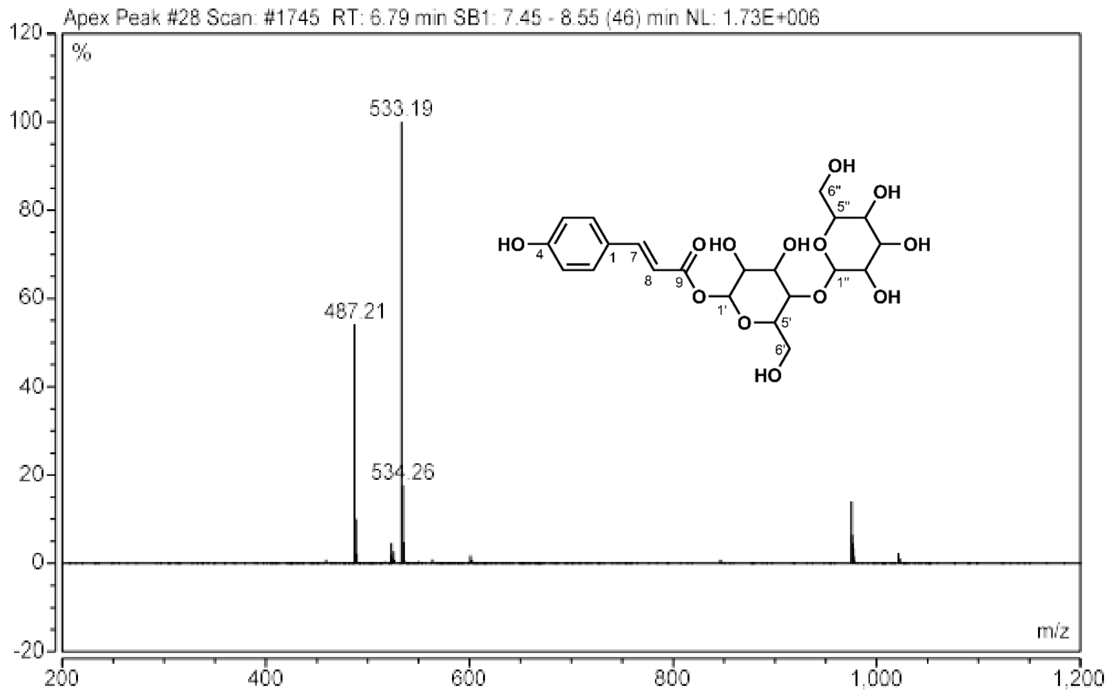

Figure S34 MS diagram of the Compound **12**

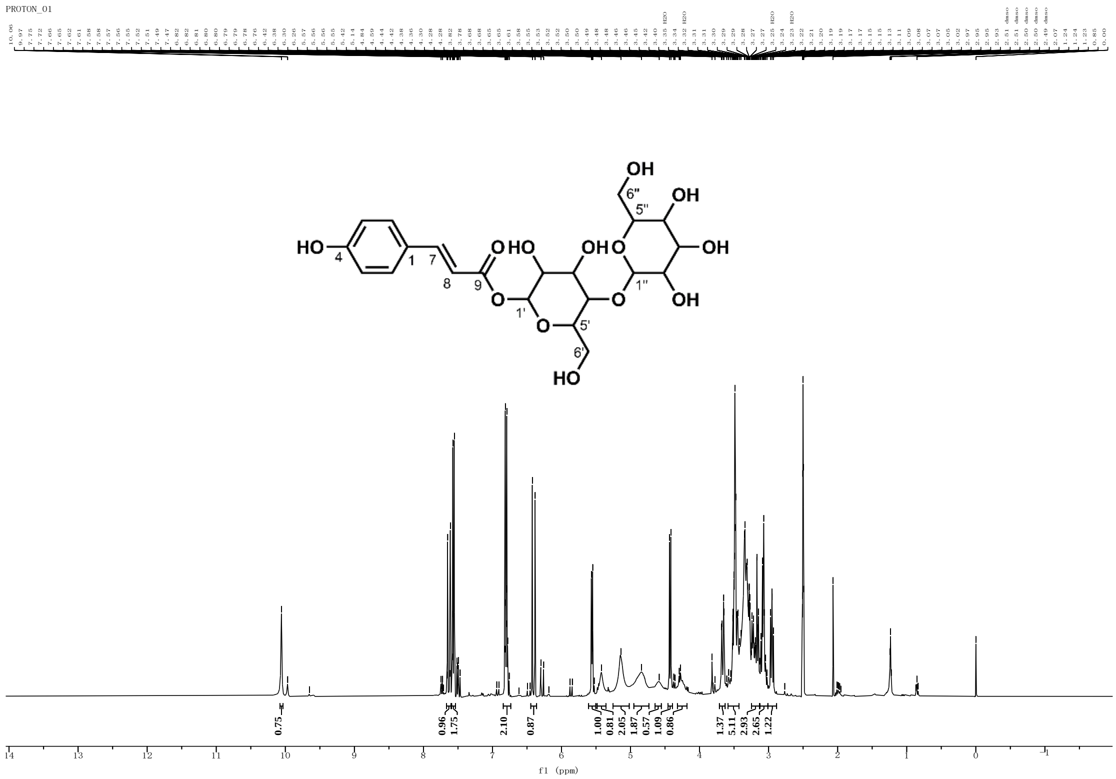

Figure S35 ^{1}H-NMR spectra of Compound **12**

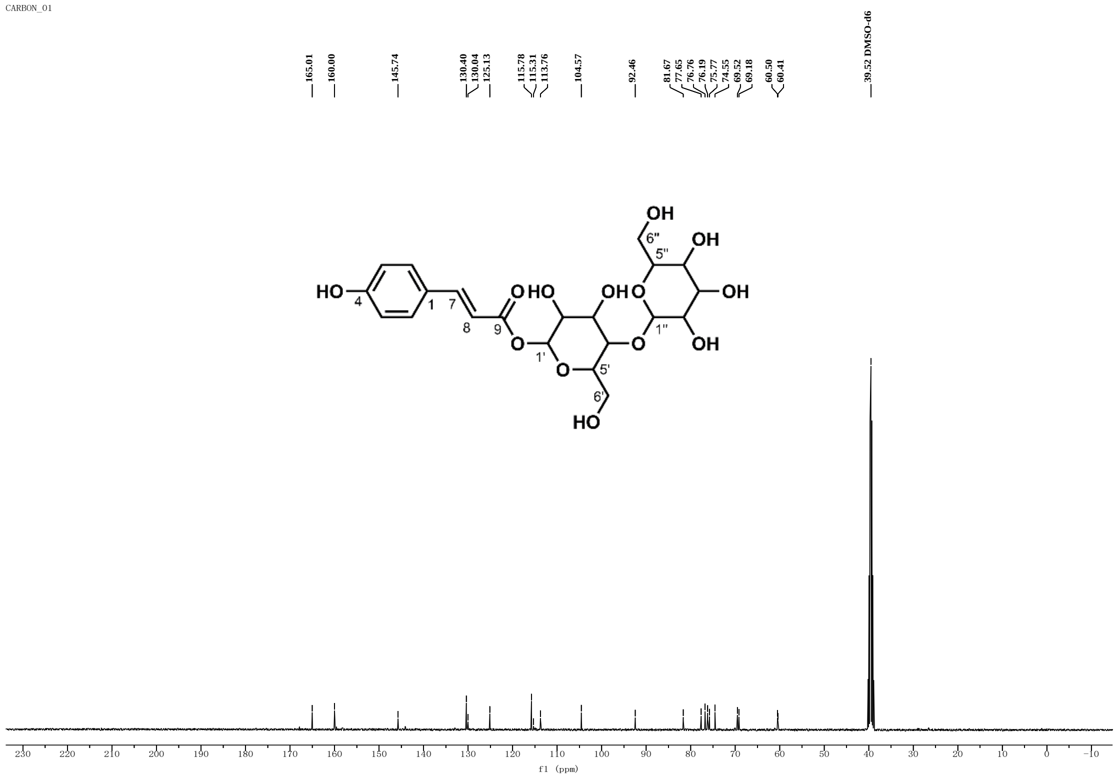

Figure S36 ^{13}C-NMR spectra of Compound **12**

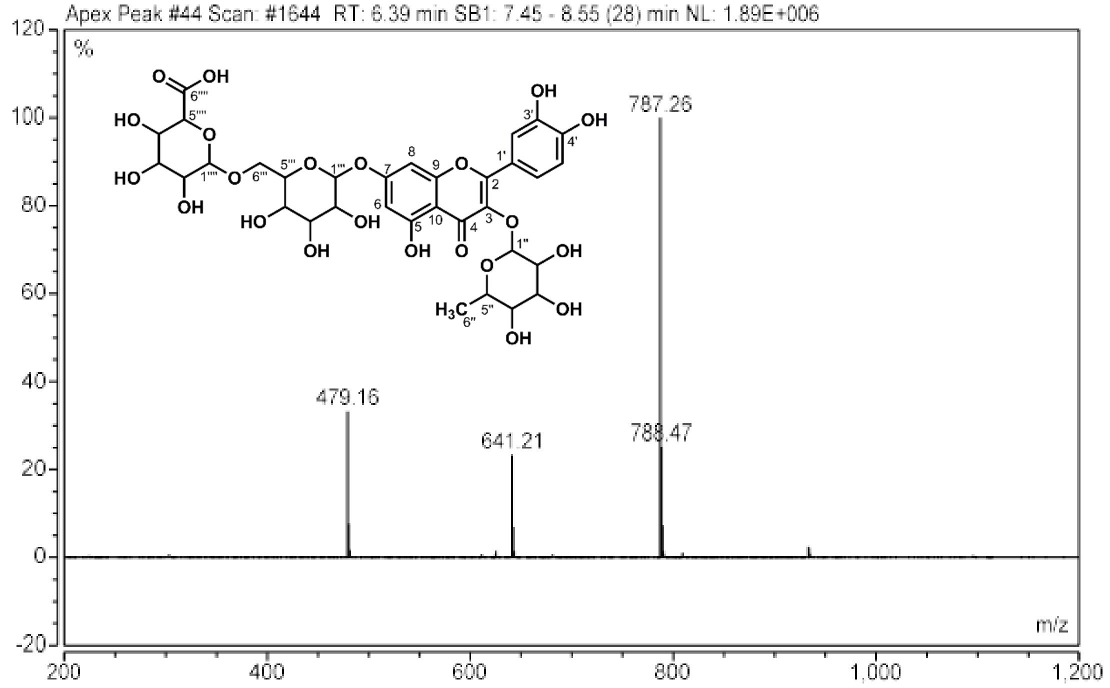

Figure S37 MS diagram of the Compound **13**

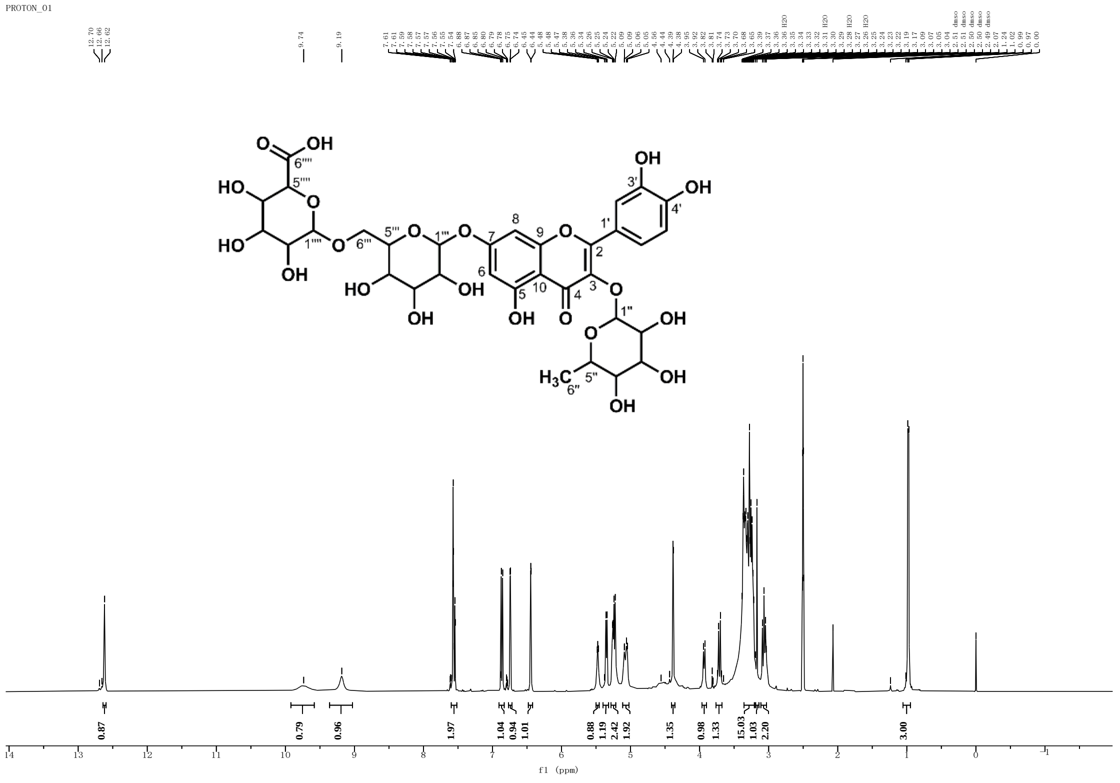

Figure S38 ^{1}H-NMR spectra of Compound **13**

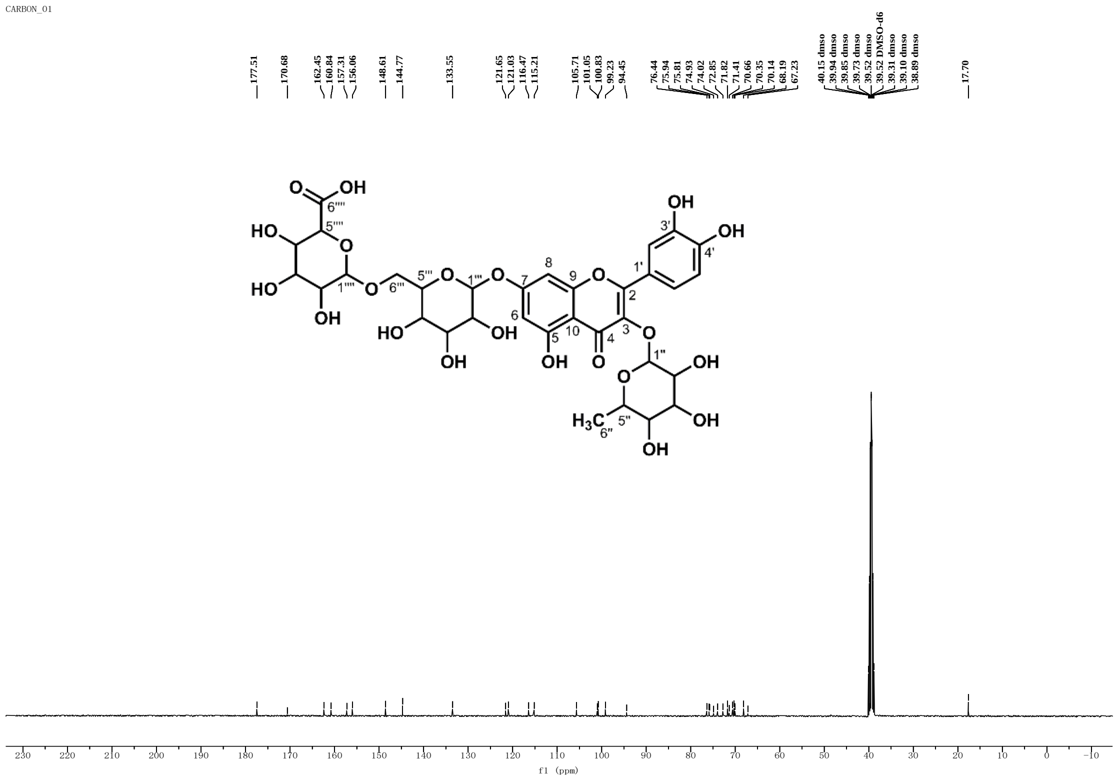

Figure S39 ^{13}C-NMR spectra of Compound **13**

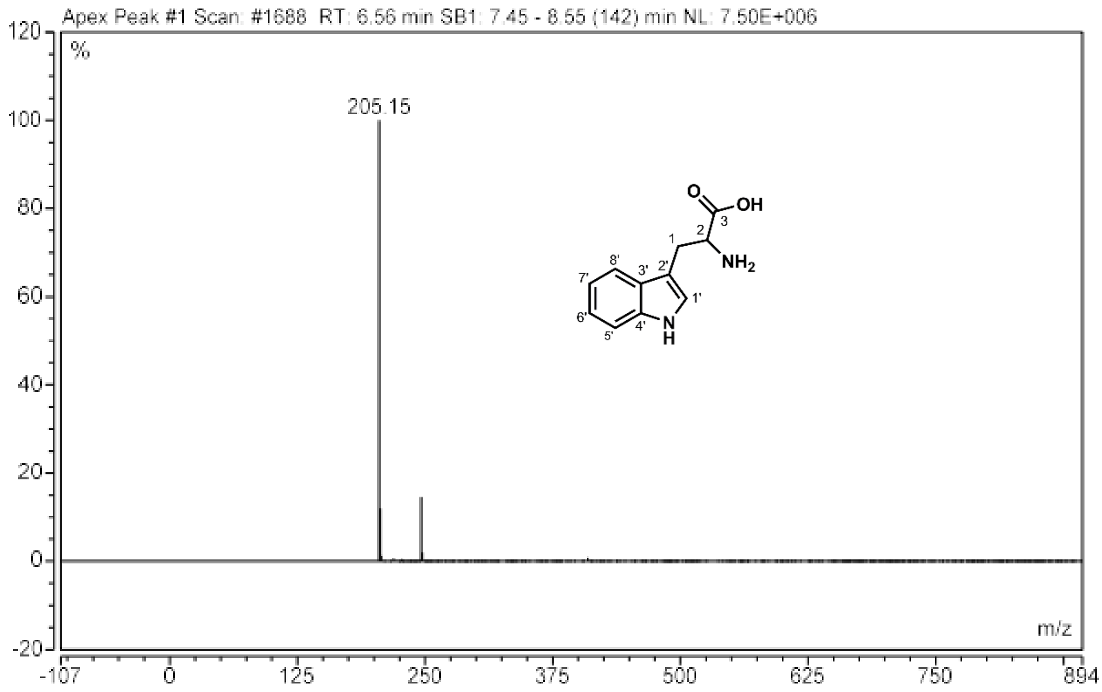

Figure S40 MS diagram of the Compound **14**

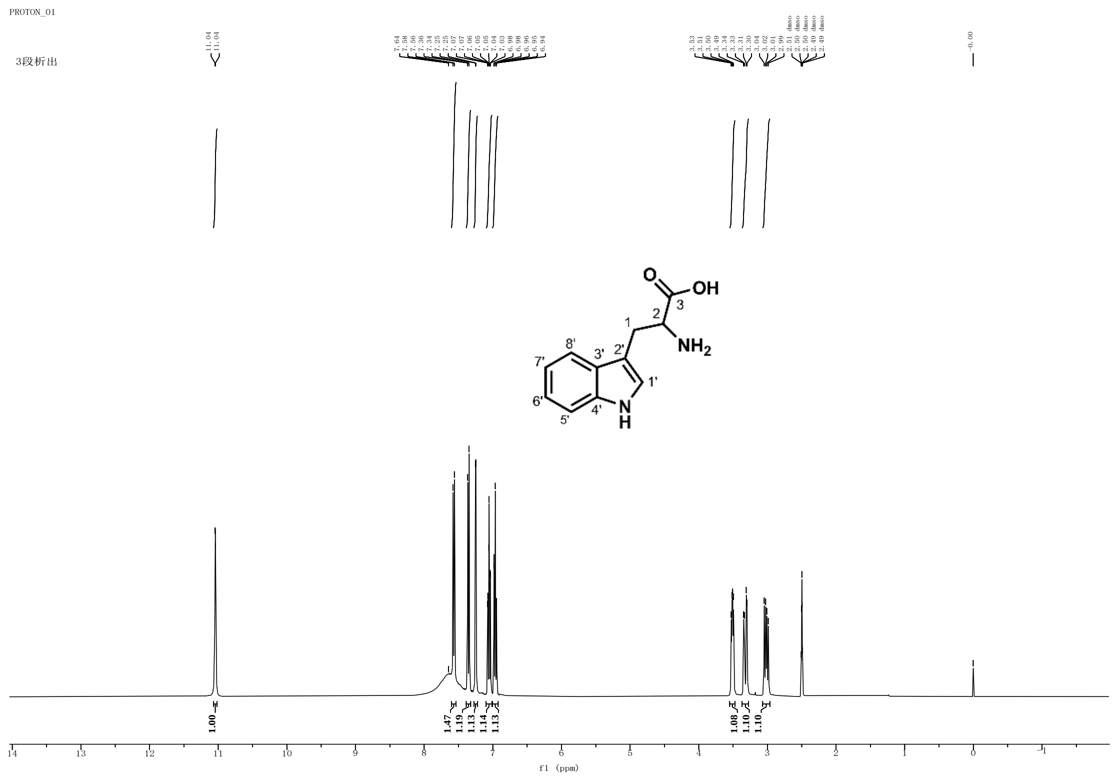

Figure S41 ^{1}H-NMR spectra of Compound **14**

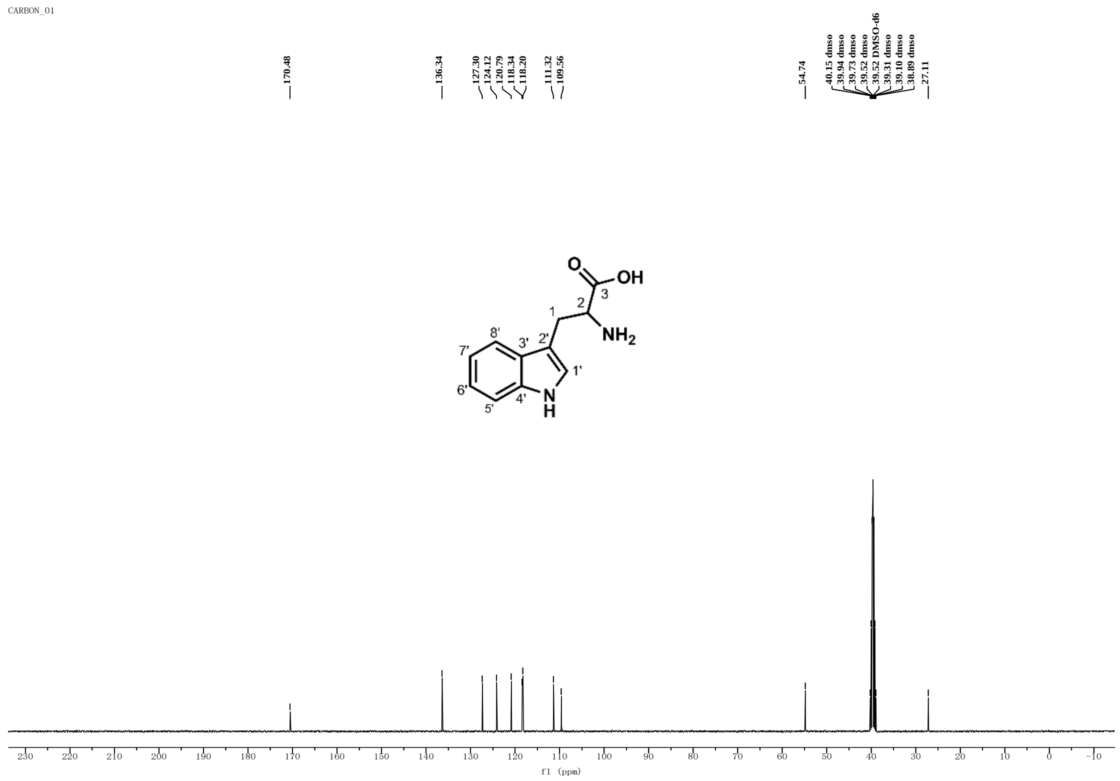

Figure S42 ^{13}C-NMR spectra of Compound **14**

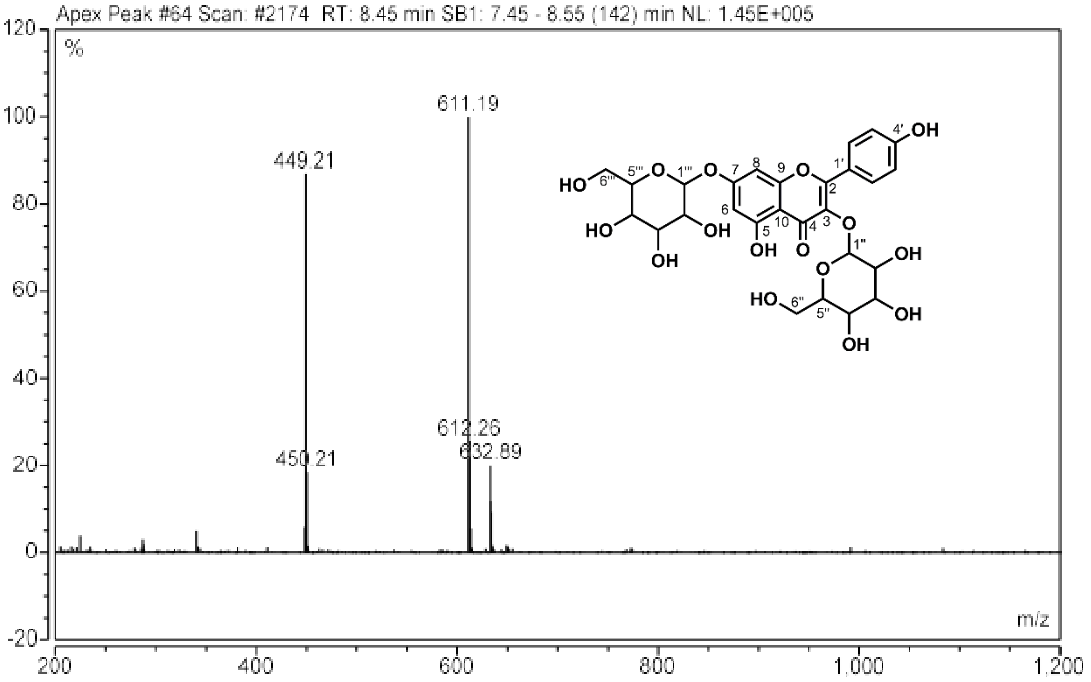

Figure S43 MS diagram of the Compound **15**

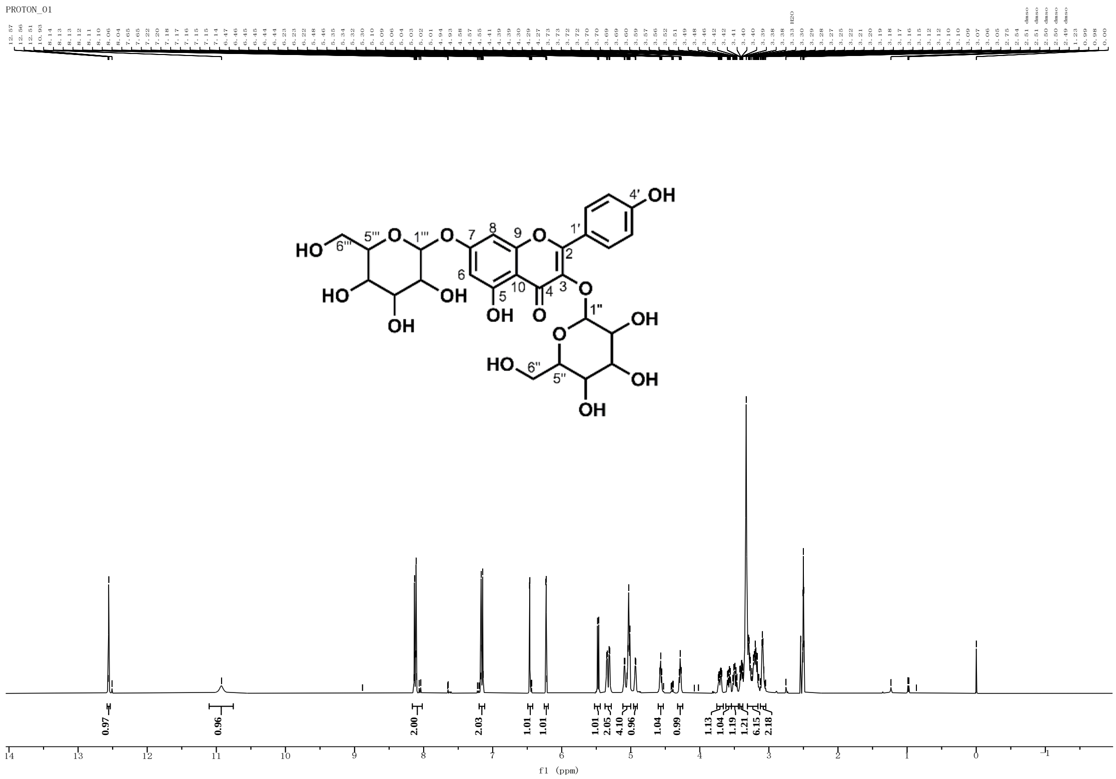

Figure S44 ^{1}H-NMR spectra of Compound **15**

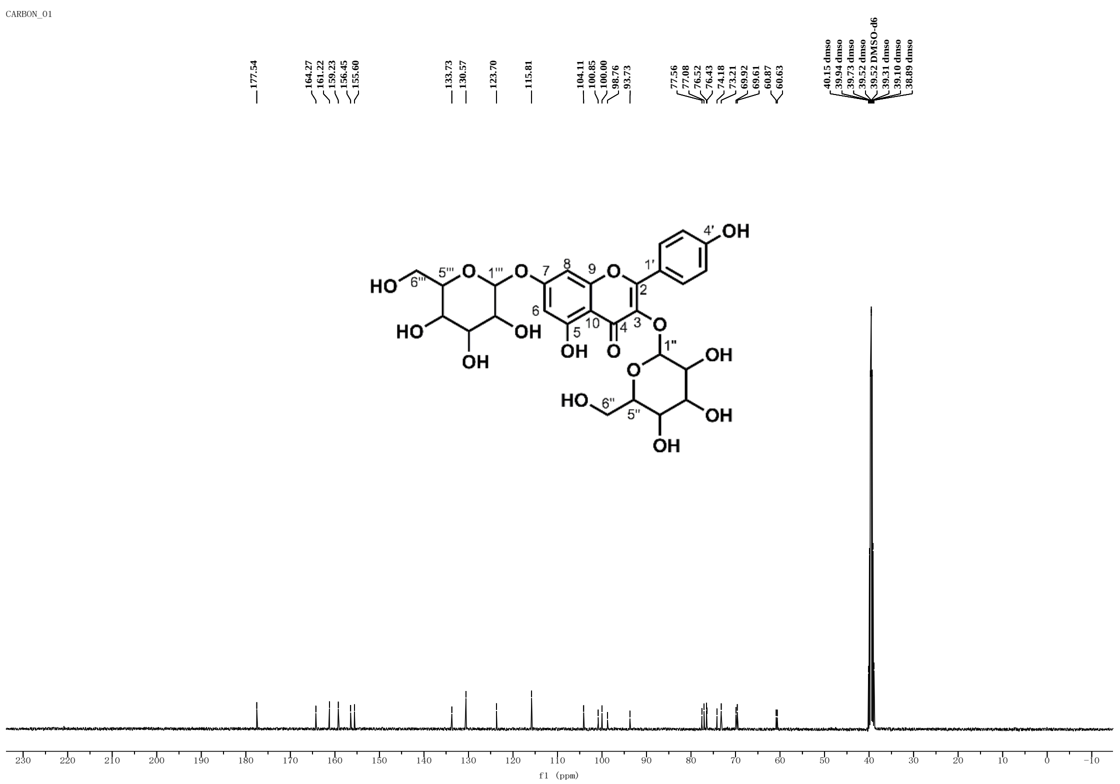

Figure S45 ^{13}C-NMR spectra of Compound **15**

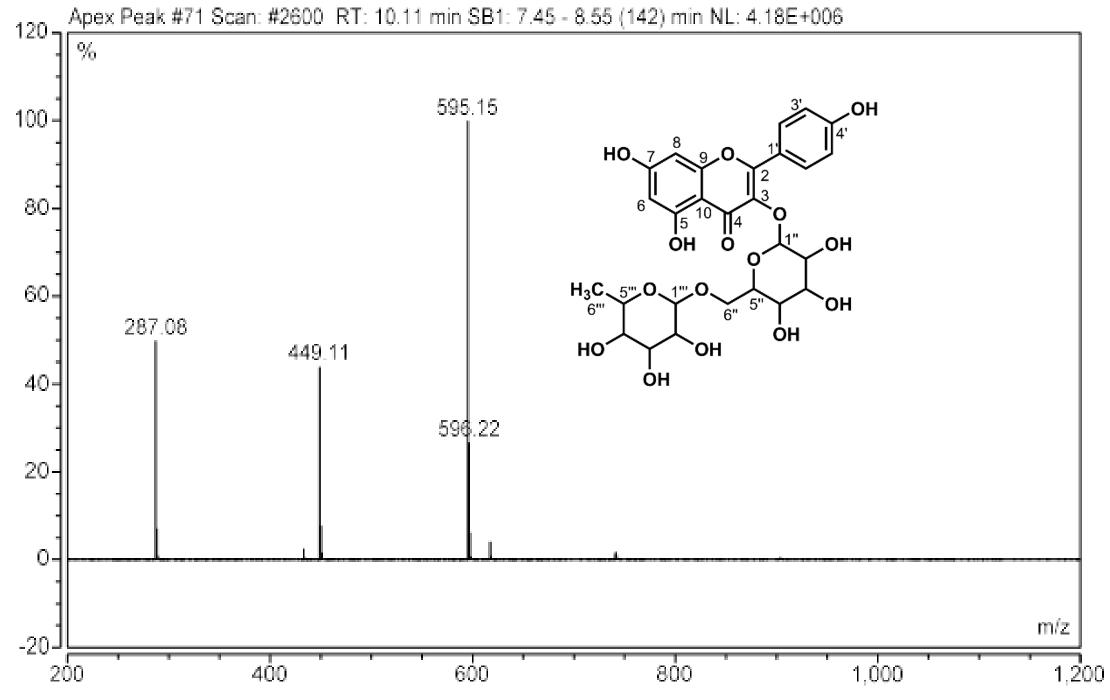

Figure S46 MS diagram of the Compound **16**

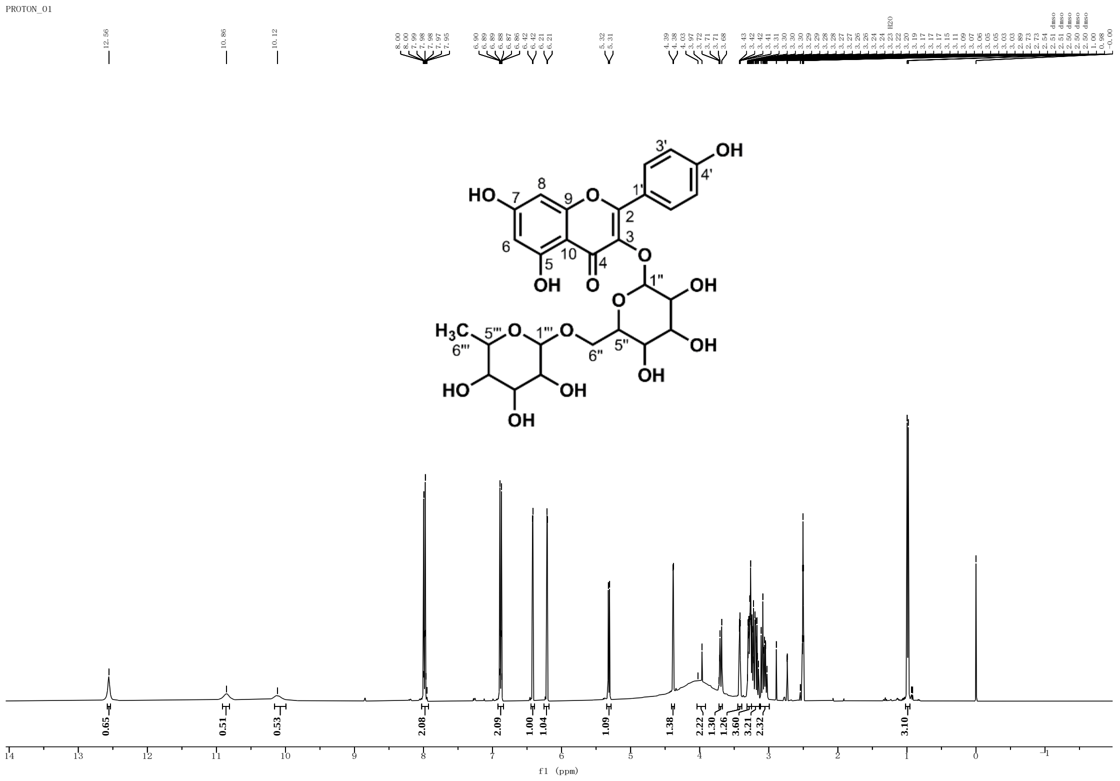

Figure S47 ^{1}H-NMR spectra of Compound **16**

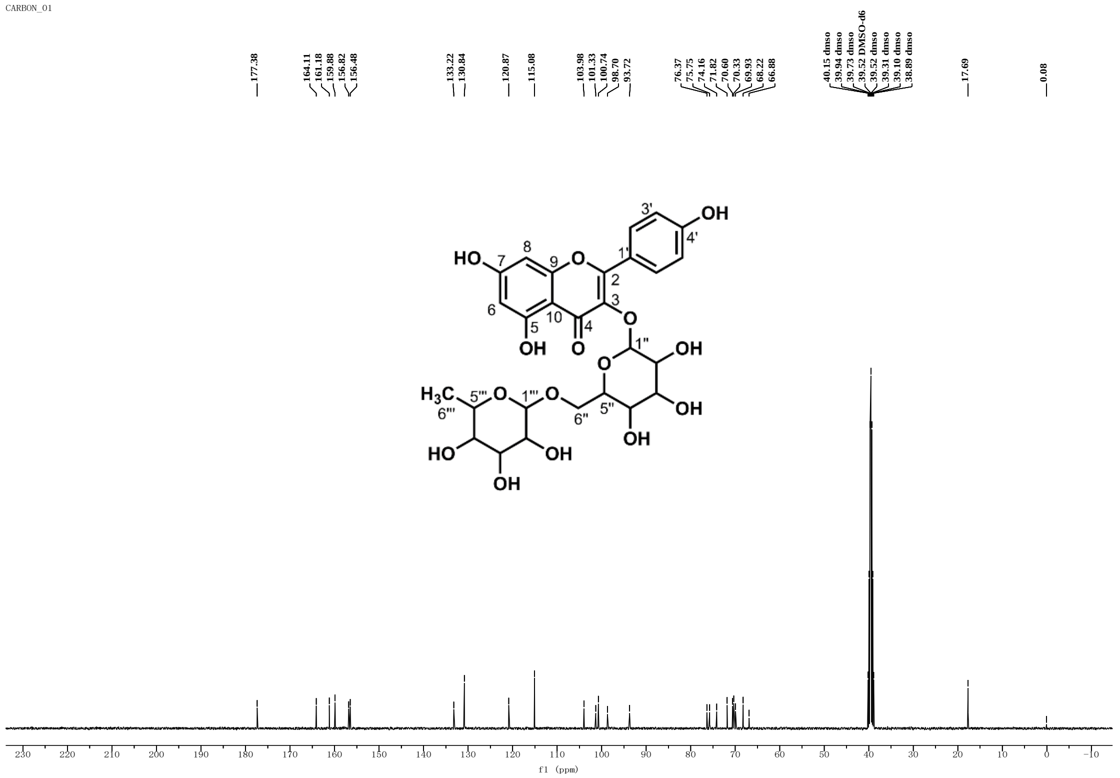

Figure S48 ^{13}C-NMR spectra of Compound **16**
